# Transcriptional Diversity in Response to Aging Across Skeletal Muscles

**DOI:** 10.1111/acel.70164

**Published:** 2025-07-09

**Authors:** Can Liu, Dongbin Zheng, Rui Zhang, Hong Li, Xingyan Tong, Yujie Wu, Geng Zhang, Siyuan Wang, Hongyu Chen, Zhinong Ren, Ying Sun, Chengdong Wang, Desheng Li, Xuewei Li, Mingzhou Li, Long Jin

**Affiliations:** ^1^ State Key Laboratory of Swine and Poultry Breeding Industry, College of Animal Science and Technology Sichuan Agricultural University Chengdu China; ^2^ Department of Geriatics Sichuan Provincial People's Hospital Chengdu Sichuan China; ^3^ Department of Geriatics, Sichuan Provincial People's Hospital, School of Medicine University of Electronic Science and Technology of Chengdu Chengdu Sichuan China; ^4^ China Conservation and Research Centre for the Giant Panda Chengdu Sichuan P. R. China

**Keywords:** aging, EGF/EGFR, myonuclei, skeletal muscles, transcriptional diversity

## Abstract

Aging leads to a gradual decline in muscle function, yet the mechanisms by which different skeletal muscles respond to aging remain unclear. Here, we constructed transcriptional maps of 11 skeletal muscles with extensive transcriptional diversity from young and old mice. Age‐related changes in gene expression displayed distinct tissue‐specific patterns, involving muscle diseases and metabolic processes. Notably, the mitochondrial‐enriched soleus muscle exhibited superior resistance to aging compared to other skeletal muscles. Further, we generated a single‐nuclei transcriptomic atlas on representative skeletal muscles, analyzing 73,170 nuclei. We found the age‐related changes in the cellular composition of different skeletal muscles and the emergence of new cell states in aged mice. Among different types of myonuclei, type II myonuclei showed particular sensitivity to aging, with reduced metabolic activity of IIb myonuclei with age. We also found cell‐specific changes occurring across nonmuscle nuclei populations, including adipocytes, fibro‐adipogenic progenitors, and immune cells, accelerating muscle aging and associated pathologies. Intercellular communication analysis revealed more intensive intercellular interactions in aged skeletal muscles, particularly between myonuclei and other cell types. Specifically, we validated the regulatory role of the EGF/EGFR axis in age‐related inflammatory processes. These findings provide insight into muscle biology and aging and highlight potential therapeutic targets for age‐associated muscle disorders.

## Graphical abstract

We constructed transcriptional maps of 11 skeletal muscles from young and old mice, revealing age‐related tissue‐specific gene expression changes. Our findings highlight cellular composition shifts and new cell states in aged muscles, providing insights into muscle aging and potential therapeutic targets for age‐related muscle disorders.

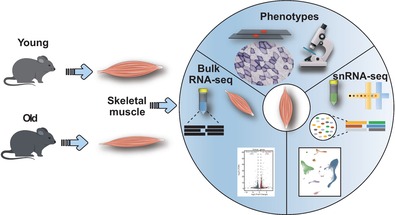

## 1 Introduction

Skeletal muscle is a major component of the animal body, accounting for approximately 40% of body weight (Florin et al. 2020; Frontera and Ochala 2014). Along with the heart, skeletal muscle contributes nearly 30% of resting energy expenditure and nearly 100% of the increased energy demand during exercise (Baskin et al. 2015; Gallagher et al. 1998). With aging, muscle mass begins to decline around middle age (approximately 1% per year) and in severe cases, up to 50% loss of muscle mass can be lost by the age of 80–90 (Wilkinson et al. 2018). This decline reduces muscle strength and physical function, a condition known as sarcopenia (Cruz‐Jentoft and Sayer 2019). Beyond impairing mobility, sarcopenia is also closely associated with metabolic dysfunction, increased inflammatory responses, and increased risk of chronic diseases (Kedlian et al. 2024).

Although transcriptomic studies have extensively examined skeletal muscle in mice, most focus on single muscles, such as the tibialis anterior (TA) and quadriceps (Bobadilla Muñoz et al. 2023; Cai et al. 2023; Kimmel et al. 2020; Kurland et al. 2023; Lin et al. 2018; Schaum et al. 2020; Zhang et al. 2022). In fact, different skeletal muscles exhibit distinct structural, functional, and developmental diversity (Terry et al. 2018). For instance, the diaphragm originates from connective tissue fibroblasts and contracts radially to drive inspiration, whereas the tibialis anterior, as a limb muscle derived from presomitic cranial mesoderm, has parallel fibers primarily responsible for lower leg movement (Merrell et al. 2015; Schiaffino and Reggiani 2011).

Aging is not uniform across muscle tissues (Gilad et al. 2024). In rats, only 2% of age‐related genes are shared across the diaphragm and hindlimb muscles (gastrocnemius, tibialis anterior, and soleus) (Shavlakadze et al. 2023). Furthermore, findings from one muscle may not apply to another (Almanzar et al. 2020; Cohen et al. 2020; Frankel et al. 2007; Schaum et al. 2020; Zhang et al. 2021). For example, aging‐induced downregulation of metabolic and mitochondrial function pathways occurs only in rat limb muscles, while extracellular matrix (ECM) pathway downregulation is primarily observed in the diaphragm (Shavlakadze et al. 2023).

In addition to structural and functional differences, skeletal muscle varies at the molecular level, with distinct fiber types displaying different aging responses (Schiaffino et al. 2024). Fast‐twitch muscle fibers, particularly type IIB, undergo significant atrophy and mitochondrial decline with aging, whereas slow‐twitch muscle fibers exhibit greater resistance to aging (Crupi et al. 2018). Thus, analyzing aging in skeletal muscle requires a tissue‐specific approach.

Skeletal muscle is a highly heterogeneous tissue composed of myofibers and diverse functionally distinct cell types, including fibroadipogenic progenitors (FAPs), adipocytes, endothelial cells, muscle stem cells (MuSCs), and various immune cells (Rodriguez et al. 2024). Prior research has identified transcriptional changes associated with aging‐related dysfunction, such as loss of myofibers and MuSCs, excessive adipocyte accumulation, adipogenic differentiation of FAPs, immune cell dysfunction, and altered intercellular communication (Giuliani et al. 2021; Jing et al. 2022; Kedlian et al. 2024; Leote et al. 2024; Li et al. 2023; Petrany et al. 2020; Wang, Zhou, et al. 2024). However, the functional decline of multinucleated muscle cells and the role of the cellular microenvironment in coordinating myofiber degradation during aging remain largely unknown.

In this study, we constructed transcriptional aging atlases across 11 anatomically distinct skeletal muscles using bulk RNA sequencing (RNA‐seq) to investigate key biological pathways involved and shared adaptive mechanisms of aging. Additionally, we employed single‐nucleus RNA sequencing (snRNA‐seq) to generate cell‐type‐resolved transcriptomic data, revealing dynamic cellular changes and potential regulatory mechanisms underlying skeletal muscle aging. Our findings highlight the critical role of the cellular microenvironment and intracellular communication in aging, including the discovery that EGF (epidermal growth factor)/EGFR ligand‐receptor‐mediated interactions between myonuclei and other cells promote inflammatory factor secretion, further driving aging. These findings provide new insights into the mechanisms of skeletal muscle functional decline and offer potential targets for future therapeutic interventions.

## 2 Results

### 2.1 Characterization of Histological Modifications in Muscle Tissues of Aging Mice

**FIGURE 1:**
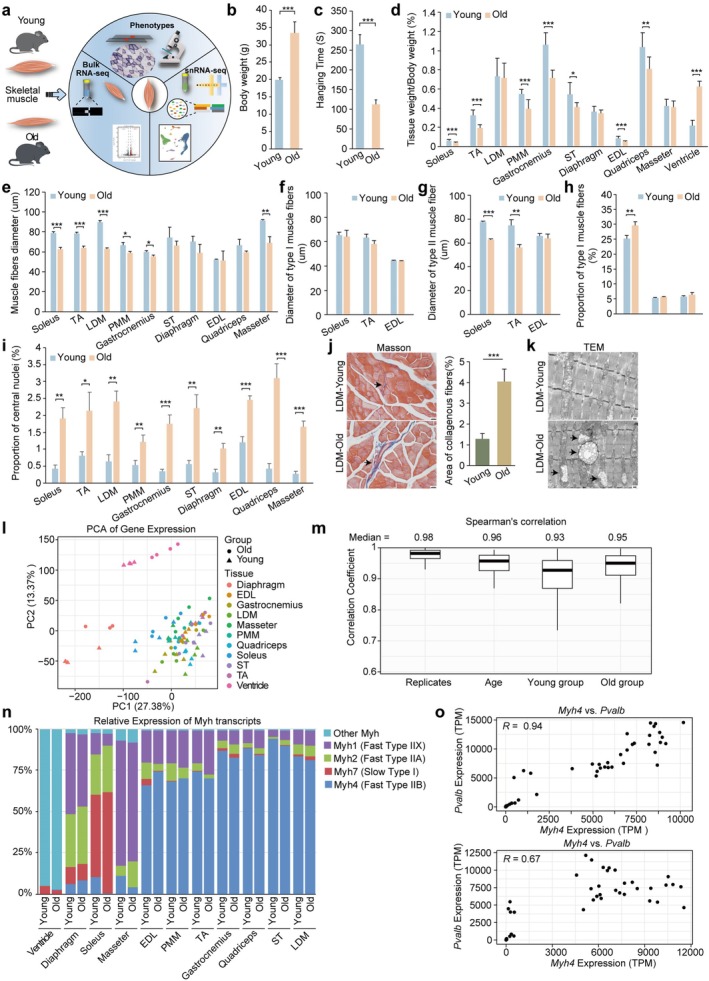
Aging‐related phenotypes and gene expression in mouse skeletal muscles. (a) Schematic representation of phenotypic analysis (*n* = 3 replicates), bulk RNA‐seq (*n* = 3 replicates), and snRNA‐seq (*n* = 2 replicates) of young and old mouse skeletal muscles. (b) Body weight of young and old mice, presented as mean ± SD. ***p < 0.001. (c) Inverted hanging time (s), presented as mean ± SD. ***p < 0.001. (d) Tissue weight‐to‐body weight ratio (%) for 11 different tissues in young and old mice, presented as mean ± SD. *p < 0.05, **p < 0.01, ***p < 0.001. (e) Muscle fiber diameter, presented as mean ± SD. *p < 0.05, **p < 0.01, ***p < 0.001. (f, g) Diameter of type I (f) and type II (g) muscle fiber, presented as mean ± SD. **p < 0.01, ***p < 0.001. (h) Proportion of type I muscle fibers, presented as mean ± SD. **p < 0.01. (i) Percentage of myofibers with central nucleus, presented as mean ± SD. *p < 0.05, **p < 0.01, ***p < 0.001. (j) Collagen area percentage in young and old mice was assessed by MASSON staining (LDM as representative). Collagen deposition indicated by arrows. Data are presented as mean ± SD. ***p < 0.001. (k) Mitochondrial morphology in young and old mice was observed by TEM (LDM as representative). Mitochondrial swelling is indicated by arrows. (l) Principal Component Analysis (PCA) of mRNA expression across 11 different tissues in young and old mice. (m) Violin plot of Spearman correlation coefficients across replicates, age groups, and within young and old mice. (n) Relative expression of Myosin heavy chain (Myh) transcripts across 11 skeletal muscle tissues in young and old mice, presented as a bar graph. The *y*‐axis represents the relative expression of Myh transcripts as a percentage of total Myh expression. (o) Expression of *Myh4* versus *Pvalb* TPM expression in young and old groups. Each dot represents one biological replicate from a tissue sample.

To elucidate the phenotypic and molecular characteristics of muscle aging, muscle tissues from young (3 months) and old (24 months) female mice (Figure 1a). A significant increase in body weight was observed in aged mice, consistent with previous studies indicating weight gain in female C57BL/6J mice during normal aging (Figure 1b) (Smith et al. 2024). A decline in the relative weight of skeletal muscle was noted, accompanied by a decrease in hanging time, indicating a gradual reduction in muscle mass and function with age, a hallmark of skeletal muscle aging (Figure 1c,d) (Börsch et al. 2021). Additionally, a significant increase in the relative weight of the heart was detected, indicating age‐related cardiac hypertrophy (Figure 1d) (Mehdizadeh et al. 2021).

SDH staining revealed a significant reduction in muscle fiber diameter in over half of the examined tissues, aligning with age‐related fiber type atrophy (Figure 1e, Figure S1a). Notably, with aging, the reduction observed in soleus (slow‐twitch) and EDL, TA (fast‐twitch) muscles decreased similarly (Figure 1d,e). Further analysis showed that the diameter of type II muscle fibers decreased significantly in soleus (*p* < 0.001) and TA (*p* < 0.01), while the diameter of type I muscle fibers decreased more in TA compared with soleus, although not significantly (Figure 1f,g). In EDL, both type I and type II muscle fibers showed a decrease, but not significantly (Figure 1f,g). Additionally, the proportion of type I muscle fibers in soleus significantly increased in the old group (*p* < 0.01) (Figure 1h). Furthermore, hallmark signs of muscle aging and degeneration were evident in all aged muscles, including an increased percentage of central nuclei observed in HE staining, muscle fat infiltration, and a significant upregulation of inflammatory and aging marker genes (Figure 1i, Figure S1b–e). Masson staining demonstrated significant collagen deposition, and electron microscopy (represented by LDM) revealed mitochondrial swelling (Figure 1j,k).

Overall, histological analysis identified multiple age‐related injury phenotypes in aging muscles, reflecting a certain degree of degeneration across all examined tissues.

### 2.2 Transcriptome Profiling Reveals Age‐Related Gene Expression in Muscles

To investigate transcriptomic alterations underlying age‐associated changes, RNA sequencing was performed on muscle tissues from young (*n* = 4) and old (*n* = 4) mice. Principal component analysis (PCA) revealed a clear distinction between the ventricle, as a cardiac muscle, and skeletal muscles (Figure 1l). Within skeletal muscles, the diaphragm, soleus, and masseter exhibited significant differences compared to other tissues, forming distinct clusters (Figure 1l, Figure S2a). Consistently, the analysis of expression level similarities (Spearman correlation coefficients) demonstrated clear differences between muscle types, confirming high consistency among biological replicates (*R* = 0.98), ensuring data reliability (Figure 1m, Figure S2a).

Skeletal muscle is composed of myofibers, which in mice are classified into four types based on *Myh* expression. It was hypothesized that the observed tissue specificity arises from differences in muscle fiber composition. To test this, the relative abundance of different fiber types was quantified by analyzing myosin heavy chain isoform expression levels (Figure 1n). Clustering analysis supported the global gene expression results, reinforcing the distinction between the ventricle and other skeletal muscle (Figure S2b). The ventricle exhibited high *Myh6* expression, which is crucial in myocardial contraction and thick filament formation in cardiac muscle (Razmara and Garshasbi 2018). Skeletal muscles with high *Myh4* expression (Type IIB myofibers) clustered together.

To further investigate these findings, marker genes for type I and type IIB myofibers were analyzed (Sadaki et al. 2023). *Pvalb*, previously identified as a type IIB myofiber‐specific marker, was found to be highly expressed in fast‐twitch skeletal muscles (Figure S2c) (Sadaki et al. 2023). Given the hypothesis that fiber type composition drives gene expression patterns, a strong correlation between *Pvalb* and *Myh4* was expected. As anticipated, a robust correlation was observed in the young group (*R* = 0.94), but this correlation significantly decreased in the old group (*R* = 0.67), suggesting that aging may affect gene regulation and fiber type composition (Figure 1o). Additionally, 24 classic type IIB myofiber markers exhibited strong correlations with Myh4 in the young group (*R* > 0.8), including Casq1 (*R* = 0.95), *Ldha* (*R* = 0.95), and *Eno3* (*R* = 0.95). However, in aged mice, 92% (22 of 24) showed reduced correlations (Figure S2d). Similarly, for slow muscle fibers, eight marker genes, such as *Myl3* (*R* = 0.95), *Atp2a2* (*R* = 0.94), and *Myoz2* (*R* = 0.92), displayed strong correlations in young mice, but seven of these showed weakened correlations in the old group (Figure S2e).

Additionally, the correlation of all expressed genes with *Myh4* and *Myh7* was calculated, identifying a representative set of fast‐ and slow‐twitch muscle fiber marker genes (Table S1). Genes strongly correlated with *Myh4* (*R* > 0.8) were primarily enriched in carbohydrate catabolic processes, whereas those correlated with *Myh7* (*R* > 0.8) were mainly enriched in muscle contraction regulation and fast‐to‐slow fiber transition (Figure S2f,g).

These findings indicate that fiber type composition plays a crucial role in maintaining gene expression patterns. However, aging significantly weakens these correlations, highlighting its impact on the regulation of fiber type‐specific gene expression.

### 2.3 Tissue‐Specific and Shared Pathways in Aging Muscle Gene Expression

**FIGURE 2:**
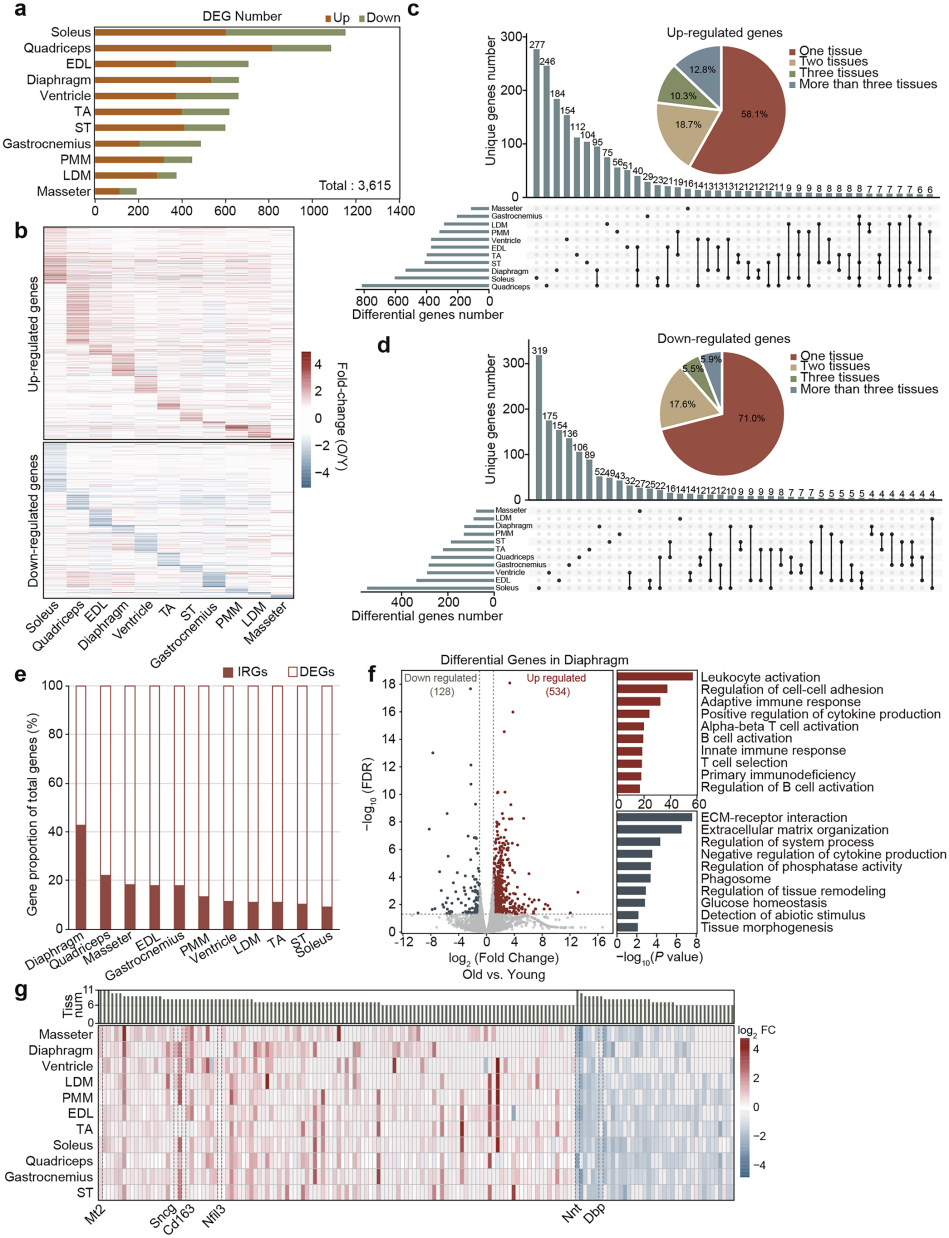
Response characterization of multiple tissues during aging. (a) Bar graph displaying the number of differentially expressed genes (DEGs) across various tissues, totaling 3615 DEGs. (b) Heatmap visualizing fold‐change values of DEGs across multiple tissues. The top panel shows upregulated genes, and the bottom panel shows downregulated genes. Each column represents a distinct tissue, with the color bar on the right representing fold‐change values (Y/O). (c, d) Percentage and upset analysis of the upregulated genes (c) and downregulated genes (d) identified in one, two, three, and more than three tissues. (e) Bar chart displaying the percentage distribution of inflammation response genes (IRGs) among differentially expressed genes (DEGs) across all tissues. (f) Left: Volcano plot showing the relative expression levels of DEGs between old and young (O/Y) mice in diaphragm. The transverse and vertical dotted lines indicate the cut‐off value for differential expression (*p* < 0.05 and abs |log_2_ fold changes| > 1). Right: Representative GO terms and KEGG pathways of upregulated and downregulated genes in diaphragm. (g) Heatmap displaying the fold change (Log_2_FC) in gene expression across different tissues. The histogram indicates the number of tissues exhibiting similar gene expression patterns.

A total of 3615 differentially expressed genes (DEGs) were identified when comparing the young and old groups (FDR < 0.05 and |log_2_fold change| > 1) (Figure 2a). Notably, the number of DEGs varied depending on anatomical location rather than muscle fiber type, with hindlimb muscles appearing to be more affected by aging.

The heatmap of fold‐change (O‐Y) for DEGs across various tissues indicates that most were identified in a tissue‐specific manner (Figure 2b). The upset analysis results demonstrate that only approximately 12.8% of the upregulated genes and 5.9% of the downregulated genes were expressed in at least three tissues, further supporting the tissue‐specific nature of aging (Figure 2c,d). This characteristic was further validated by the functional enrichment analysis of genes uniquely expressed in different skeletal muscle regions, such as oxidative phosphorylation enrichment in soleus, skeletal muscle contraction in TA, and inorganic ion transmembrane transport in ventricle (Figure S3a–k).

However, functional enrichment analysis revealed that most upregulated genes were enriched in inflammation and immunity pathways across multiple tissues. Notably, 10 pathways were consistently enriched in at least six tissues, including inflammatory response, cytokine‐cytokine receptor interaction, and regulation of cell–cell adhesion (Figure S4a). These findings align with previous quantitative results of the inflammatory marker *Il‐1b*, with all tissues exhibiting varying degrees of inflammatory damage. Interestingly, the diaphragm had the highest proportion of inflammation response genes (IRGs), such as *Ccl3* and *Tbx21*, which are associated with respiratory inflammation (Figure 2e). Consistently, immunofluorescence staining showed a significant upregulation of TNF‐α‐positive nuclei in the old diaphragm group (Figure S4b). Pathway enrichment analysis of the diaphragm revealed that the upregulated genes were significantly enriched in immune response‐related pathways, including pro‐inflammatory factors like *Ifng* and *Irf8* (Figure 2f). GSEA analysis yielded consistent results, with the Th17 cell differentiation pathway, which primarily secretes pro‐inflammatory cytokines, being significantly upregulated, while the ECM pathway was downregulated in the diaphragm (Figure S4c,d).

In contrast, the enriched pathways of downregulated genes were more tissue‐specific and related to growth and development (Figure S4e). Nonetheless, we observed that ECM organization pathways were significantly downregulated in more than half of the tissues, indicating a widespread decline in tissue regeneration and repair capacity with aging.

Consistently, multiple genes exhibited a similar pattern of change across different tissues (Figure 2g). For example, *Nnt*, a crucial mitochondrial redox regulatory protein that promotes chronic inflammation associated with aging and metabolic diseases, was significantly downregulated in all 11 tissues (Figure 2g). Conversely, *Mt2*, which induces various types of skeletal muscle atrophy, was significantly upregulated in the same tissues (Figure 2g). Other genes, including *Dbp* (a circadian rhythm factor), *Cd163* (an inflammation marker), *Nfil3* (an immune regulator), and *Sncg* (a muscle atrophy factor), also showed significant changes in the majority of skeletal muscle regions (Figure 2g).

In summary, aging exerts widespread effects across all tissues. Changes in gene expression exhibit significant tissue specificity, with a greater number of differential genes observed in hindlimb muscles exhibiting rather than being predominantly characterized by muscle fiber type. Additionally, certain shared biological pathways, such as inflammatory responses, are activated. Notably, the diaphragm, as the primary respiratory muscle, exhibits a more pronounced inflammatory response.

### 2.4 Tissue‐Specific Alterations of Muscle Disease‐Related Genes During Aging in Different Skeletal Muscles

Most research treats skeletal muscles as a homogenous group, often overlooking the significant differences in gene expression profiles. However, the genetic heterogeneity of these muscle tissues may determine their unique responses to aging and related diseases. Therefore, examining tissue‐specific gene expression changes across skeletal muscles during aging is essential for understanding the onset and progression of skeletal muscle diseases.

Sarcopenia, a common age‐related pathological condition, is characterized by progressive and generalized muscle loss, resulting in a decline in muscle mass and strength (Antuña et al. 2022; Grima‐Terrén et al. 2024). In rodent models, sarcopenia is typically assessed in hindlimb muscles to investigate gene expression changes associated with disease progression (Sayer et al. 2024). However, it remains unclear whether muscles from different anatomical regions are equally affected and whether the underlying mechanisms are consistent. To address this, 659 sarcopenia‐related genes were analyzed across 11 skeletal muscles during aging (Liang et al. 2023; Pannerec et al. 2016). Among these, 189 genes exhibited significant alterations in at least one muscle, with an average of 37 genes per muscle (Table S2). The greatest number of DEGs was observed in the soleus and quadriceps, with 61 and 58 genes, respectively, while the masseter exhibited the fewest, with 12 genes (Figure S5a). These findings align with previous studies indicating that hindlimb muscle mass declines more rapidly with age and that sarcopenia, often associated with neuromuscular dysfunction, initially manifests in the hindlimbs of rats (Pannerec et al. 2016; Sayer et al. 2024).

Notably, only 10 genes exhibited significant alterations in at least 6 tissues, with consistent gene expression patterns of change (upregulation or downregulation) across multiple tissues. For example, *Aplnr*, a potential therapeutic target for age‐related muscle atrophy, was significantly downregulated in the diaphragm, gastrocnemius, longissimus dorsi muscle (LDM), masseter, psoas major muscle (PMM), quadriceps, semitendinosus (ST), and soleus, suggesting a role in muscle atrophy during aging (Vinel et al. 2018).

More than half of the DEGs (103 of 189, or 54.5%) exhibited tissue specificity, with alterations observed in only one muscle. For instance, neuromuscular junction (NMJ) degeneration during aging leads to denervation, a key mechanism underlying sarcopenia, with fast‐twitch muscles being the most affected (Cedric et al. 2014). *Chrnd*, a gene related to NMJ denervation, was significantly upregulated in the TA, a representative fast‐twitch muscle, in the old group.

Myokines play a crucial role in muscle aging by regulating inflammatory responses and intercellular signaling, impacting both local and systemic aging processes (Lightfoot and Cooper 2016). In aged muscle, myokine secretion is frequently altered, contributing to inflammation, muscle atrophy, and metabolic dysregulation (Mancinelli et al. 2021). Of the 578 identified myokines, 141 exhibited significant changes in at least one skeletal muscle (Table S3). Hindlimb muscles displayed the highest number of differentially expressed myokine‐related genes, with 53 in quadriceps and 40 in soleus (Figure S5b). In contrast, trunk muscles exhibited fewer alterations, with 27 in PMM, 20 in LDM, and 19 in masseter (Figure S5b). The findings indicate that hindlimb muscles, which require greater strength and endurance and frequently engage in high‐intensity activity, may be subject to distinct regulatory mechanisms influencing myokine production.

Among these differentially expressed myokines, only 16 genes showed significant changes across at least six tissues, exhibiting consistent differential expression trends. These included several chemokines, such as *Ccl5*, *Ccl6*, *Ccl8*, and *Ccl9*. Conversely, nearly half (70 of 141, or 49.65%) of the differentially expressed myokines were unique to a single muscle. For example, *Hspb1* and *Hspb6*, which are involved in distal myopathies characterized by lower limb muscle weakness, were significantly downregulated in TA (Ranta‐Aho et al. 2024).

To further investigate skeletal muscle gene expression in age‐related muscle diseases, 389 genes associated with abnormal skeletal muscle cell development were analyzed, with 121 linked to dystrophy, 106 to atrophy, 60 to abnormal morphology, 53 to myopathy, and 34 to Inflammation (Qiu et al. 2023; Terry et al. 2018). Among these, 171 genes exhibited significant changes in at least one muscle tissue during aging (Table S4), including 53 genes associated with abnormal skeletal muscle cell development, 37 with dystrophy, 34 with atrophy, 19 with myopathy, and 14 each with inflammation and abnormal morphology.

Hindlimb muscles displayed the highest number of DEGs, with 57 in soleus and 51 in quadriceps, compared to 20 in LDM and 19 in masseter (Figure S5c). Although soleus exhibited the greatest number of DEGs, nearly half were downregulated, including *Mstn*. Co‐IF staining revealed consistent results, showing that *Mstn* is expressed only in type IIA muscle fibers, and its expression was significantly downregulated in the old group (Figure S5d). This decline in Mstn expression reflects the loss of muscle mass during aging, and our findings suggest that in soleus, this loss is predominantly derived from type IIA muscle fibers (Fan et al. 2017).

Interestingly, 78 of 171 DEGs (45.61%) showed significant changes in a single muscle. For instance, *Myh14* mutations can lead to progressive muscle weakness, predominantly impacting distal limb muscles (Choi et al. 2011). In our analysis, *Myh14* was significantly upregulated in TA. Trunk muscles also displayed distinct gene expression profiles; for example, *Hpgds*, a gene implicated in DMD and associated with immune‐mediated skeletal muscle damage, was significantly downregulated in PMM (Hamamura et al. 2024; Okinaga et al. 2002).

In summary, skeletal muscles exhibit unique gene expression changes during aging, leading to varying susceptibilities to muscle diseases. These findings underscore the necessity of developing muscle‐specific treatment strategies rather than applying generalized therapeutic approaches.

### 2.5 Distinct Metabolic Features During Aging in Different Muscle Tissues

Energy metabolism is crucial for muscle function, with different muscle fiber types exhibiting distinct metabolic characteristics based on their functional demands. Slow‐twitch muscle fibers primarily generate ATP through oxidative phosphorylation, whereas fast‐twitch muscle fibers rely predominantly on glycolysis (Schiaffino et al. 2024).

To further investigate metabolic differences across muscle tissues during aging, the expression of genes involved in key metabolic pathways was analyzed. These included glycolysis (45 genes), oxidative phosphorylation (109 genes), fatty acid oxidation (78 genes), the tricarboxylic acid (TCA) cycle (31 genes), and insulin response (189 genes). Significant differences in gene expression patterns were observed between muscles with a high proportion of type IIB muscle fibers and those with a low proportion of type IIB muscle fibers, such as diaphragm, ventricle, soleus, and masseter (Figure S5e–i).

Aging was found to significantly alter the expression of 12 glycolysis‐related genes, 20 oxidative phosphorylation genes, 9 fatty acid oxidation genes, 1 TCA cycle gene, and 28 insulin response‐related genes in at least one skeletal muscle tissue (Table S5). Notably, only a few genes were significantly altered across multiple tissues (≥ 6), including glycolysis‐related genes (*Pfkfb3*, *Gck*), oxidative phosphorylation genes (*mt‐Nd3*, *Snca*), fatty acid oxidation genes (*Lep*), and insulin response‐related genes (*Inhbb*, *Prkcz*). The majority of changes (40 out of 70, or 57.1%) were specific to a single muscle.

For example, *Ucp2*, a key glycolysis‐related gene, has been associated with reduced ATP production in skeletal muscle, leading to muscle fatigue and decreased exercise capacity (Yin et al. 2021). Upregulation of *Ucp2* was observed in quadriceps, a muscle‐rich type IIB muscle fiber, suggesting its potential role in age‐related metabolic alterations affecting muscle mass.

In contrast, genes associated with oxidative phosphorylation were significantly upregulated in the soleus, a slow‐twitch muscle (Figure S5j). These included *Cox8a*, *Cox5b*, *Cox5a1*, *Ndufb3*, *Cox5a2*, *Uqcr11*, and *Ndufa1*. The distinct upregulation of these genes in the soleus was confirmed through pathway enrichment analysis (Figure S5k). Previous studies have demonstrated that oxidative fiber‐enriched muscles, such as the soleus, exhibit superior mitochondrial homeostasis and greater resistance to mitochondrial aging compared to glycolytic fiber‐rich muscles, such as the TA (Crupi et al. 2018; Elmansi and Miller 2024).

Compared to other skeletal muscles, the soleus displayed the most pronounced changes in mitochondrial homeostasis‐related genes, with a greater number of DEGs and a substantial upregulation pattern (Figure S5l). These alterations were accompanied by a significant increase in slow‐twitch muscle fibers and a corresponding decrease in fast‐twitch muscle fibers (Figure S5m). Additionally, several muscle disease‐associated genes, including *Pdp1* and *Bub1b*, were significantly downregulated in the soleus compared to other skeletal muscles.

These findings underscore the distinct metabolic features of muscle tissues with varying fiber compositions, highlighting the superior mitochondrial stability and resistance to aging observed in oxidative fiber‐rich muscles, such as the soleus.

### 2.6 SnRNA‐Seq Identifies Age‐Related Changes in Cell Types and Gene Expression in Skeletal Muscle

**FIGURE 3:**
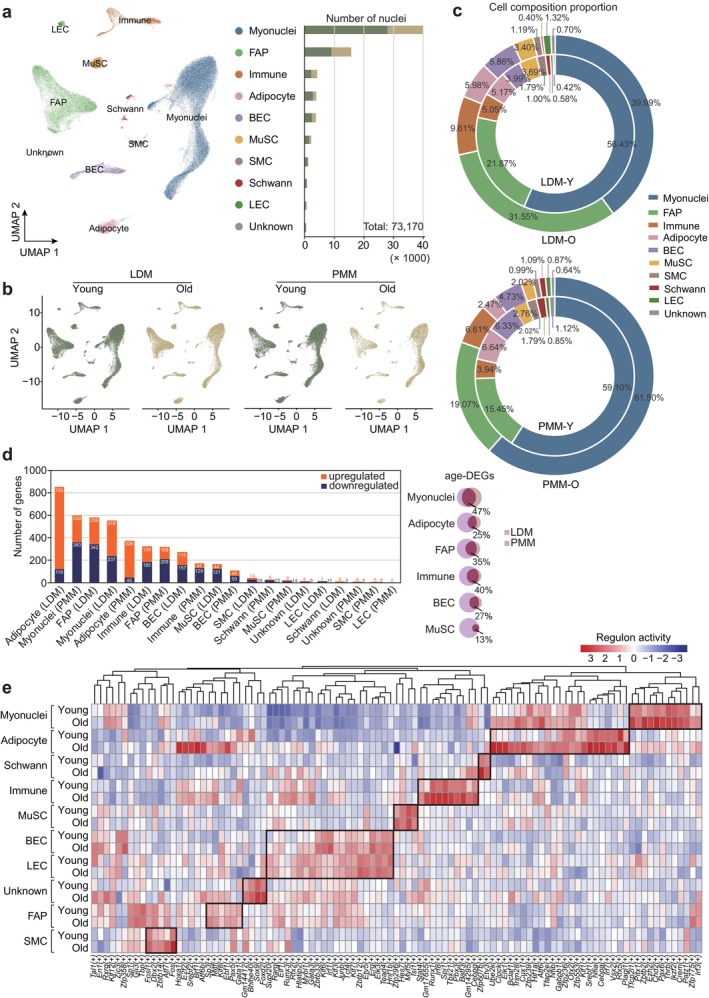
Single‐nucleus transcriptomic atlas of mouse skeletal muscle aging. (a) Uniform Manifold Approximation and Projection (UMAP) diagram of young and old skeletal muscle (left). Cell types include myonuclei, fibro‐adipogenic progenitors (FAPs) immune cells, adipocytes, blood vascular endothelial cells (BECs), MuSCs, smooth muscle cells (SMCs), Schwann cells (Schwann), lymphatic endothelial cells (LECs), and unknown nuclei (unknown). The number of nuclei for each cell type is shown (right). (b) UMAP diagram displaying nuclear clusters across different tissues in young and old groups. (c) Pie charts showing the proportional composition of nuclear clusters in LDM (left) and PMM (right) for young and old groups. (d) The number of upregulated and downregulated DEGs across all cell clusters in LDM and PMM. Cell types are ranked by the total number of DEGs (left). Venn diagrams indicate the percentage of common DEGs among specific cell types from different tissues. Circle size is proportional to the number of DEGs (right). (e) Heatmap of transcription factor (TF) activity predicted by SCENIC analysis for all cell types for young and old groups.

To compare cell populations in young and old skeletal muscle, single‐nucleus RNA sequencing (snRNA‐seq) was performed on LDM and PMM. After quality control and filtering, 73,170 high‐quality single‐nucleus transcriptomes were retained from eight individual libraries (Figure S6a,b). Using uniform manifold approximation and projection (UMAP) analysis, several major populations were identified, each exhibiting distinct transcriptome signatures. The cellular origin of the nuclei was determined based on marker gene expression, classifying them as myonuclei, FAPs, immune cells, adipocytes, blood vascular endothelial cells (BECs), MuSCs, smooth muscle cells (SMCs), Schwann cells, lymphatic endothelial cells (LECs), and unidentified nuclei (Figure 3a, Figure S6c). The top 15 signature genes for each cell type are provided in Figure S6d.

Cellular composition alterations between young and old groups in LDM and PMM were subsequently analyzed. Myonuclei were identified as the predominant cell type, with non‐muscle populations, including FAPs, immune cells, and adipocytes also constituting a significant proportion of the total cell population (Figure 3b,c, Figure S6e). Despite aging, the fundamental cell type composition in LDM and PMM remains largely unchanged.

To clarify aging‐associated gene expression changes at the cellular level, DEGs were identified across all major cell types. Aging had the most pronounced effects on adipocytes (LDM = 854, PMM = 371), myonuclei (LDM = 553, PMM = 599), FAPs (LDM = 581, PMM = 318), and immune cells (LDM = 323, PMM = 173) (Figure 3d, left). A comparative analysis of DEGs across the same cell types in different tissues revealed a shared DEG proportion ranging from 13% to 47%, suggesting that age‐related transcriptional alterations in the same cell types followed similar biological pathways (Figure 3d, right). Functional enrichment analyses identified pathways such as muscle cell differentiation (myonuclei), regulation of cold‐induced thermogenesis (adipocytes), ECM organization (FAPs), and leukocyte activation (immune cells) (Figure S6f,g). However, the muscle system process pathway was consistently downregulated across all cell types.

Single‐cell regulatory network inference and clustering (SCENIC) analysis was conducted to assess transcription factor activity across cell types. A total of 101 active regulons were identified in both young and old groups (Figure 3e). Based on regulon activity scores, nuclei were classified into their expected types, and cell type‐specific transcription factors were identified. For instance, *Pitx1*, which is involved in myogenic differentiation, was enriched in myonuclei; *Cebpa*, associated with lipid synthesis, was enriched in adipocytes; and *Tbx21*, involved in immune response, was enriched in immune cells (Fatemi Langroudi et al. 2023; Knopp et al. 2013; Lau et al. 2023; Sara et al. 2019).

These results indicate that aging significantly impacts gene expression in adipocytes, myonuclei, and FAPs, affecting distinct biological pathways and transcription regulatory networks.

### 2.7 Heterogeneity of Myonuclei in Response to Aging

**FIGURE 4:**
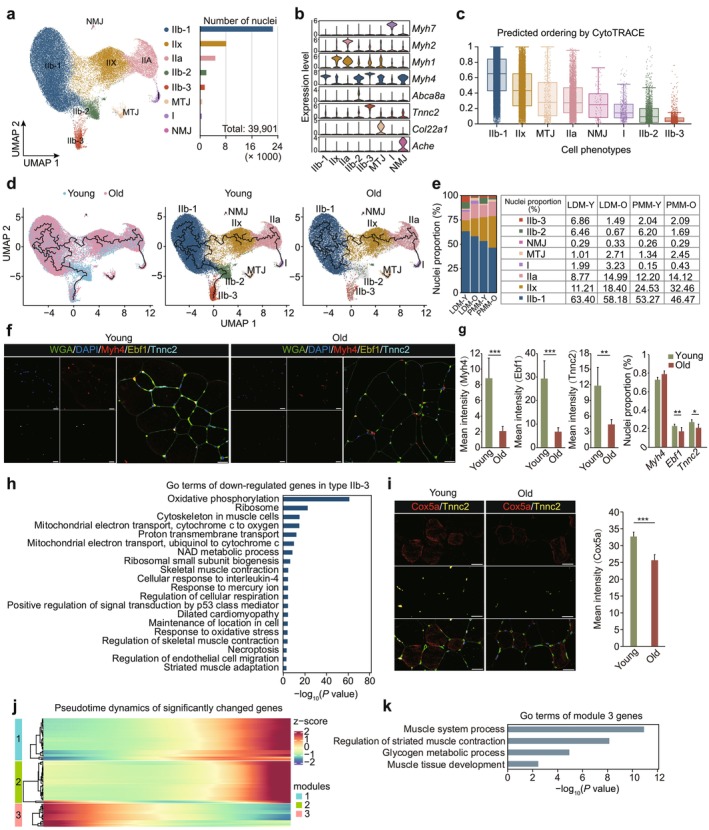
Reclustering and pseudotemporal trajectories identify transcriptional dynamics of myonuclei. (a) Uniform Manifold Approximation and Projection (UMPA) diagram of myonuclei subclusters in young and old skeletal muscle (left). Subclusters include IIb‐1, type IIb‐1 myonuclei; IIx, type IIx myonuclei; IIa, type IIa myonuclei; IIb‐2, type IIb‐2 myonuclei; IIb‐3, type IIb‐3 myonuclei; MTJ, myotendinous junction myonuclei; I, type I myonuclei; NMJ, neuromuscular junction nuclei. The number of nuclei in each subcluster is shown (right). (b) Violin plots showing the expression of marker genes across different clusters. (c) Box plots illustrate the differentiation potential score of each cell type (range: 0–1), where higher scores indicate greater differentiation potential and a less differentiated state. (d) UMAP visualization of myonuclear trajectory in young and old groups (left). Myonuclei are color‐coded by subtype (middle and right). (e) Proportion of each myonuclei subcluster in young and old groups. (f) Representative immunostaining images of marker genes for type IIb‐1 (Myh4), IIb‐2 (Ebf1), and IIb‐3 (Tnnc2) in young and old groups. Scale bar = 20 μm. (g) Quantification of marker gene immunofluorescence intensities and nuclei proportion presented as mean ± SD (right). *p < 0.05, **p < 0.01, ***p < 0.001. (h) Gene ontology (GO) terms and KEGG pathways of downregulated genes in type IIb‐3 myonuclei. (i) Representative immunostaining images of Cox5a and Tnnc2 in the young and old groups are shown on the left. Scale bar = 20 μm. Gene immunofluorescence intensities were quantified and presented as mean ± SD on the right. ***p < 0.001. (j) Pseudotemporal heatmap of gene expression dynamics for significantly altered marker genes. (k) GO terms and KEGG pathways of genes in module 3.

Among all total clusters, myonuclei constituted the majority. To explore their heterogeneity, we further reclustered them and identified eight distinct subclusters: type I myonuclei (expressing *Myh7*), type IIa (*Myh2*), type IIx (*Myh1*), myotendinous junction (MTJ, *Col22a1*), and NMJ (*Ache*) (Figure 4a,b, Figure S7a). Notably, type IIb was further divided into three subclusters: IIb‐1 (*Myh4*), IIb‐2 (*Abca8a*), and IIb‐3 (*Tnnc2*) (Figure 4b). Functional enrichment analysis of marker genes showed that IIb‐1 was related to skeletal muscle development, IIb‐2 was significantly enriched in phosphatidylinositol‐mediated signaling, and IIb‐3 was enriched in oxidative phosphorylation (Figure S7b–d).

To examine the trajectory changes of different myonuclei subtypes with aging, we performed pseudotime trajectory analysis using Monocle 3. Cytotrace analysis revealed a continuous trajectory beginning with type IIb, progressing through type IIx and type IIa, and concluding with type I (Figure 4c, Figure S7e). Interestingly, type IIb subclusters followed a dynamic trajectory from IIb‐1 to IIb‐2 and then to IIb‐3 (Figure 4d). However, in the old group, this trajectory was disrupted, with the transition from IIb‐1 to IIb‐3 absent. This disruption was accompanied by a significant reduction in type IIb‐2 and IIb‐3 myonuclei, suggesting that aging impairs the dynamic trajectory of type IIb‐1 into type IIb3 (Figure 4d,e, Figure S7f). Immunostaining of marker genes in young and old muscles confirmed their expression in myofibers. The proportion of type IIb‐1 myonuclei remained unchanged, whereas the type IIb‐2 and IIb‐3 myonuclei were significantly downregulated, with significantly reduced mean fluorescence intensity in the old group (Figure 4f,g).

To further investigate the mechanisms underlying myonuclei subtype responses to aging, we analyzed DEGs in each cluster. Compared to type I myonuclei, type II myonuclei exhibited a higher number of DEGs, suggesting a susceptibility to aging (Figure S7g). Muscle atrophy markers *Fbxo32* and *Trim63* were notably upregulated in type II fibers (Sartori et al. 2021). Notably, all myonuclei subtypes displayed significant enrichment in RNA splicing pathways, indicating that dysregulated RNA splicing may drive muscle fiber aging (Figure S7h–m).

Previous studies have linked high oxidative phosphorylation activity to mitochondrial homeostasis and protection against skeletal muscle decline (Crupi et al. 2018). However, we observed a significant downregulation of oxidative phosphorylation pathways in type IIb‐3 myonuclei, including key genes such as *Chchd10*, *Park7*, and *Cox5a*, which are essential for maintaining mitochondrial structure and function (Figure 4h). Mutations in these genes have been linked to various neuromuscular disorders (Skou et al. 2024; Xiao et al. 2020). Co‐immunofluorescence staining of Tnnc2 with Cox5a confirmed this finding, with the mean intensity of Cox5a (a subunit of mitochondrial complex IV) significantly reduced in the old group (Figure 4i). Consistent with these findings, pseudotime dynamics of significantly altered genes revealed that downregulated module 3 was associated with muscle system processes, suggesting that oxidative phosphorylation downregulation contributes to muscle function decline, particularly in type IIb‐3 fibers during aging (Figure 4j,k).

Taken, our findings demonstrate that myonuclear subtypes undergo substantial changes during aging. Type II myonuclei are more susceptible to aging, with inhibited transition of IIb‐1 myonuclei into IIb‐3 and concurrent downregulation of oxidative phosphorylation pathways in IIb‐3 myonuclei.

### 2.8 Transcriptional Changes in Nonmuscle Nuclei During Aging

Multiple non‐myonuclear cell populations contribute to the skeletal muscle microenvironment. To explore the role of nonmuscle nuclei in the aging muscle microenvironment, the major cell types, including adipocytes, FAPs, immune cells, and MuSC, were classified into distinct subtypes.

**FIGURE 5:**
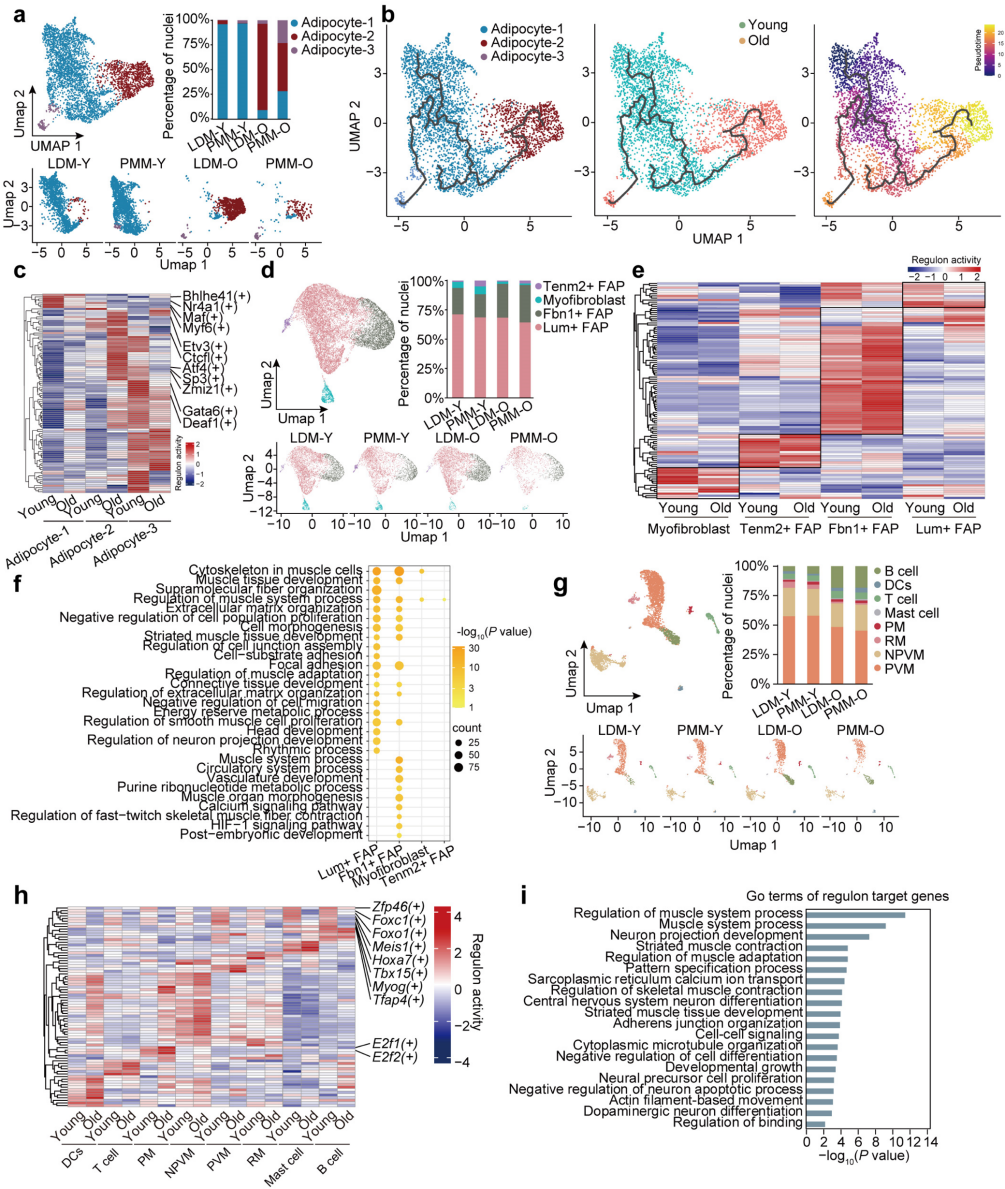
Reclustering reveals transcriptional dynamics of nonmuscle nuclei. (a) Umap diagram of adipocyte subclusters from young and old skeletal muscle (top left). The proportion of each adipocyte subcluster in different groups (top right). The UMAP diagram visualizes nuclear clusters of different tissues in the young and old groups (bottom). (b) UMAP showing the trajectory of adipocyte subclusters obtained from young and old groups (left). The nuclei are colored according to groups (middle) and pseudotime (right). (c) Heatmap of gene activity of transcription factors (TFs) predicted by SCENIC analysis of adipocyte subclusters in young and old groups. (d) UMAP diagram of FAP subclusters from young and old skeletal muscle (top left). The proportion of each FAP subcluster in different groups is shown (top right). UMAP diagram visualizes nuclear clusters of different tissues in the young and old groups (bottom). (e) Heatmap of gene activity of TFs predicted by SCENIC analysis of the FAP subcluster for young and old groups. (f) Dot plot of GO terms and KEGG pathways enriched among DEGs in the FAP subcluster. (g) UMAP diagram of immune cell subclusters from young and old skeletal muscle (top left). The proportion of each immune cell subcluster in different groups is shown (top right). The UMAP diagram visualizes nuclear clusters of different tissues in the young and old groups (bottom). (h) Heatmap of gene activity of TFs predicted by SCENIC analysis of immune cell subclusters for young and old groups. (i) GO terms and KEGG pathways of selected TF target genes in the immune cell subcluster.

Adipocytes were subdivided into three distinct subpopulations: adipocyte 1, associated with lipid homeostasis (*Fasn*‐positive); adipocyte 2, related to cell adhesion (*Bcl2*‐positive); and adipocyte 3, involved in energy metabolism and thermogenesis (*Ucp1*‐positive) (Figure 5a, Figure S8a–d). Trajectory analysis indicated high plasticity among adipocytes, with adipocyte 1 serving as the dynamic trajectory starting point, branching into adipocyte 2 and adipocyte 3 (Figure 5b). Immunofluorescence staining confirmed changes in nuclear ratios, revealing significant downregulation of Abca8 in the old group, while Bcl2 and Ucp1 were significantly upregulated (Figure S8e,f). GSEA analysis demonstrated consistent results, with aging inducing upregulation of adherens junction and autophagy pathways in adipocyte 2, while fatty acid metabolism and PPAR signaling pathways were enriched in adipocyte 3 (Figure S8g,h). Based on pseudotime dynamic expression patterns, downregulated module 2 was associated with muscle system processes, whereas module 3 was linked to cell adhesion, apoptosis, and lipid accumulation (Figure S8i,j). Transcription factor activity scoring classified all adipocytes into the expected subtypes (Figure 5c). Distinct transcription factors exhibited high activity within each subtype. In adipocyte 1, the activity of transcription factors related to adipocyte development (*Nr4a1*) and muscle fiber maturation (*Bhlhe41*, *Maf*, *Myf6*) was enhanced in the old group. In adipocyte 2, transcription factors associated with neurodegenerative diseases, type 2 diabetes, and cancer (*Ctcfl*, *Etv3*, *Atf4*, *Zmiz1*, *Sp3*) showed reduced activity in the old group (Figure 5c). In adipocyte 3, transcription factors associated with brown fat formation (*Gata6*, *Deaf1*) exhibited increased activity in the old group (Figure 5c). These findings indicate that adipocytes exhibit substantial plasticity. With aging, lipid synthesis and metabolism‐associated adipocyte 1 transitions into adipocyte 2, which is characterized by enhanced cell adhesion pathways and upregulation of transcription factors linked to neurodegenerative diseases.

FAPs, mesenchymal progenitor cells located in the interstitial region of skeletal muscle, play a critical role in muscle maintenance, regeneration, and growth (Giuliani et al. 2021). The most abundant subtypes, *Lum +* FAP and *Fbn1+* FAPs, were identified, consistent with previous findings in the quadriceps and diaphragm of mice (Rubenstein et al. 2020). Based on marker gene expression, FAPs were classified into four subtypes: *Tenm2+* FAP, myofibroblasts, *Fbn1+* FAP, and *Lum +* FAP (Figure 5d and S9a). The trajectory analysis revealed that in normal muscle, a continuous trajectory extends from *Fbn1*+ FAP to *Lum* + FAP, with two branches forming at the distal end, which differentiate into *Tenm2+* FAP and myofibroblasts, respectively (Figure S9b). No significant changes in nuclear proportions were observed during aging (Figure 5d). Transcription factor activity profiles aligned with clustering results, confirming these classifications (Figure 5e). Immunofluorescence staining of marker genes remained largely unchanged, except for Fbn1, which was significantly upregulated in the old group (Figure S9c,d). However, functional enrichment analysis of DEGs revealed alterations in pathways such as muscle tissue development, ECM organization, and cell morphogenesis, predominantly enriched in *Lum +* FAP and *Fbn1+* FAP (Figure 5f). These findings suggest that *Lum +* and *Fbn1+* FAPs, as key components in maintaining muscle structure and function, undergo more pronounced changes during aging. Excessive ECM accumulation‐induced fibrosis leads to muscle failure and tissue dysfunction, ultimately leading to mortality (Borthwick et al. 2013; Henderson et al. 2020; Pakshir and Hinz 2018; Smith and Barton 2018). Therefore, *Lum +* and *Fbn1*+ FAP may play a potential role in age‐related muscle pathology.

Re‐clustering of immune cells identified eight distinct subpopulations, including four macrophage subtypes: perivascular‐like macrophages (PVMs), non‐perivascular‐like macrophages (NPVMs), proliferative macrophages (PMs), and regulatory macrophages (RMs) (Figure 5g, Figure S9e). The remaining four subpopulations were annotated as B cells, T cells, dendritic cells (DCs), and mast cells (Figure 5g, Figure S9e). Macrophages, the predominant immune cells in skeletal muscle, exhibited a decline in NPVMs, PVMs, and RMs (Figure 5g). Despite these shifts, the immunofluorescence staining intensity of marker genes remained largely unchanged (Figure S9f,g). Notably, DEG analysis of PVMs and NPVMs revealed significant enrichment in immune response activation pathways (Figure S9h). Transcription factor activity analysis supported the classification, with distinct transcription factors identified within each cluster. *E2f1* and *E2f2*, key regulators of the cell cycle, exhibited unique activation in PMs (Figure 5h). Interestingly, several transcription factors—including *Zfp46*, *Foxc1*, *Foxo1*, *Meis1*, *Hoxa7*, *Tbx15*, *Myog*, and *Tfap4—*were suppressed across all cell types in the old group (Figure 5h). Functional enrichment analysis revealed that target genes were significantly enriched in the muscle system process, suggesting that transcriptional changes in immune cells during aging may affect skeletal muscle function and maintenance (Figure 5i).

In addition, the re‐clustering of MuSCs highlighted the heterogeneity of MuSCs and identified two subpopulations: MuSC quiescent (*Pax7*‐positive) and MuSC activated (*Acta1*‐positive) (Figure S9i,j). The proportion of these subpopulations did not show significant changes (Figure S9i). Functional enrichment analysis revealed that the downregulated genes in MuSC activated were significantly enriched in pathways related to mitochondrial homeostasis, suggesting that with aging, MuSC activated may undergo a mitochondrial aging process (Figure S9k).

In conclusion, non‐muscle cell populations undergo significant transcriptional changes during aging, contributing to altered interstitial microenvironment in aged muscle. These changes may accelerate muscle aging and associated pathologies.

### 2.9 EGF/EGFR Signaling Pathway Mediates Inflammation During Cellular Aging

**FIGURE 6:**
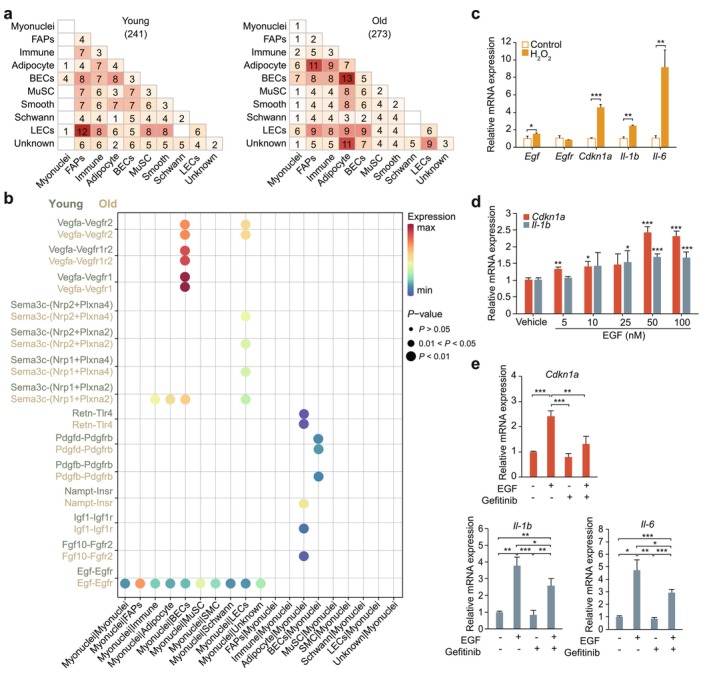
Potential ligand‐receptor interactions were analyzed between myonuclei and other cell types. (a) Heatmap showing the number of inter‐population communications in young (left) and old (right) groups. (b) Ligand‐receptor pairs showing significant changes in specificity between young and old groups, with myonuclei acting as a ligand or receptor in interactions with other populations. (c) Relative expression of *Egf*, *Egfr*, *Cdkn1a*, *Il‐1b*, and *Il‐6* validated by qRT‐PCR in control and H_2_O_2_‐treated cells. Data are presented as mean ± SD. *p < 0.05, **p < 0.01, ***p < 0.001. (d) Relative expression of inflammatory (*Cdkn1a*) and aging (*Il‐1b*) marker genes validated by qRT‐PCR in the cells treated with EGF at different concentrations for 3 days. Data are presented as mean ± SD. *p < 0.05, **p < 0.01, ***p < 0.001. (e) Relative expression of *Cdkn1a*, *Il‐1b*, and *Il‐6* validated by qRT‐PCR in the control group and after treatment with EGF, Gefitinib, or both.

Skeletal muscle, as an endocrine organ, is capable of producing and secreting various cytokines. To investigate how the intercellular crosstalk in the skeletal muscle niche is affected by aging, intercellular communication patterns mediated by ligand‐receptor interactions were analyzed using CellChat (Jin et al. 2021). Overall, ligand‐receptor interactions predominantly involved mononucleated cells rather than myofibers (Figure 6a, Figure S10a). For example, the secretion of the Igf1 ligand by adipocytes and its interaction with the Igf1r receptor on other cell types were enhanced in the old group, an interaction previously linked to the induction of adipocyte differentiation (Figure S10b) (D'Esposito et al. 2012; Han et al. 2017; Xu and Liao 2004).

With aging, the total number of interactions increased, particularly in myonuclei, where the percentage of interactions increased nearly fourfold, indicating more intensive intercellular communication in aged skeletal muscle (Figure S11a,b). Compared to the young group, aged myonuclei exhibited a significant increase in interactions between the Egf ligand and the Egfr receptor (Figure 6b). Studies have demonstrated that EGF is the primary ligand for EGFR, and EGFR activation induces cellular senescence while promoting the secretion of inflammatory cytokines through the Ras signaling pathway (Shang, Han, et al. 2020). This aligns with the upregulation of inflammatory pathways observed at the tissue level. These findings suggest that aging enhances the secretion of EGF from myonuclei, which interacts with EGFR, a transmembrane receptor distributed on the surface of other cell types, to induce the expression of inflammatory cytokines in the tissue.

To verify EGF expression changes in aging muscle fibers, C2C12 cells were treated with 485 μM H_2_O_2_ to establish a cellular aging model. Compared to the control group, the H_2_O_2_‐treated group exhibited reduced cell differentiation and smaller myotube formation (Figure S11c). Quantitative results showed that *Egf*, *Cdkn1a*, *Il‐1b*, and *Il‐6* were significantly upregulated in the H_2_O_2_ group, while Egfr remained unchanged (Figure 6c). These findings confirm the successful establishment of the aging model and the upregulation of Egf and inflammatory markers.

To further investigate the mechanism by which EGF induces inflammatory factor expression, C2C11 cells were treated with different concentrations of EGF (0, 5, 10, 25, 50, and 100 nM). As shown in Figure 6d, the expression levels of *Cdkn1a* and *Il‐1b* were highest at 50 nM EGF. Compared to the vehicle, the expression of *Cdkn1a* and *Il‐1b* nearly doubled, indicating that 50 nM EGF significantly induces senescence in C2C11 cells.

Gefitinib, a small‐molecule EGFR inhibitor, was used to investigate the interaction between EGF and EGFR. Compared to the control group (EGF‐ + Gefitinib‐), the addition of EGF alone significantly upregulated *Cdkn1a*, *Il‐1b*, and *Il‐6* gene expression (Figure 6e). However, this upregulation was significantly inhibited when EGF and gefitinib were simultaneously added (Figure 6e). Interestingly, the addition of gefitinib alone resulted in a significant downregulation of *Il‐1b* expression compared to the control group (Figure 6e). Additionally, the myogenic factor *Myog* was quantified, showing the opposite trend to inflammatory markers (Figure S11d). EGF alone significantly inhibited *Myog* expression, while the simultaneous addition of EGF and gefitinib significantly increased *Myog* expression (Figure S11d). These results suggest that EGF not only regulates inflammatory responses through the EGFR signaling pathway but may also influence the expression of muscle growth factors.

Overall, the study results demonstrated that EGF plays a crucial role in the aging process by activating the EGFR signaling pathway. EGF induced the expression of the cellular senescence marker *Cdkn1a* and the inflammatory factor *Il‐1b* while inhibiting the expression of the muscle growth factor Myog. The EGFR inhibitor gefitinib partially reversed these effects, further confirming the key regulatory role of the EGF/EGFR axis in aging‐related inflammation.

## 3 Discussion

Aging‐induced skeletal muscle atrophy is characterized not only by a reduction in muscle mass but also by fiber‐type switching, diminished muscle contractile function, and increased inflammation (Marzetti et al. 2024). Previous studies have often used a single skeletal muscle as a representative of the entire muscle family (Terry et al. 2018). However, more than 50% of transcripts are differentially expressed across skeletal muscles, indicating that distinct skeletal muscle groups exhibit unique aging responses (Terry et al. 2018). This variation underscores the need to investigate site‐specific changes in skeletal muscles to fully understand the mechanisms underlying muscle‐related diseases.

As expected, fiber‐type composition, specifically the proportion of fast‐twitch and slow‐twitch muscle fibers, contributes to the observed diversity in gene expression. Consistent with previous studies, ventricular (cardiac) muscle differs significantly from skeletal muscle, while skeletal muscle tissues with higher *Myh4* expression cluster more closely together (Terry et al. 2018). However, DEG analysis did not reveal a clear trend associated with muscle fiber classification and instead appeared to be largely tissue‐specific, particularly in the hindlimb muscles, which exhibited a greater number of differentially expressed genes. A similar pattern is observed in Duchenne muscular dystrophy (DMD), where contraction‐induced injuries primarily affect the calf and respiratory muscles, including the soleus, gastrocnemius, and diaphragm (Crupi et al. 2018; Denti et al. 2006; Klingler et al. 2012; Tsuda 2018; Villalta et al. 2014). Notably, the soleus, a slow‐twitch muscle, demonstrates a stronger anti‐aging capability. Specifically, although the diameters of both type I and type II muscle fibers decrease similarly compared to fast‐twitch muscles, the proportion of type I muscle fibers significantly increases. Such compensatory shifts in fiber composition may help preserve mitochondrial respiration in the soleus muscle, consistent with studies suggesting that oxidative skeletal muscles better maintain mitochondrial function during aging due to fiber composition and enhanced mitochondrial homeostasis (Elmansi and Miller 2024; Rodriguez et al. 2024).

By comparing multiple skeletal muscle tissues from young (3‐month‐old) and old (24‐month‐old) mice, we observed age‐related damage phenotypes across all muscle groups. Aging skeletal muscles exhibit varying degrees of inflammatory responses, with the diaphragm displaying a particularly pronounced inflammatory response among 11 muscles analyzed (Pan et al. 2021). Recent studies suggest that fast/glycolytic fibers tend to express inflammation‐related genes associated with aging (Zhang et al. 2024). Given that diaphragm IIx/IIb muscle fibers primarily function in short‐term, high‐intensity activities such as airway clearance, the selective atrophy of these fibers may reduce airway clearance capacity (Fogarty et al. 2019; Greising et al. 2013). We hypothesize that this decline leads to an accumulation of foreign particles, pathogens, and inflammatory mediators, thereby exacerbating inflammation‐related gene expression in the diaphragm. It is important to emphasize that the correlations observed between gene expressions in different pathways do not imply causation or direct transcriptional linkage. Further experimental validation, such as functional assays, will be necessary to confirm these relationships.

snRNA‐seq revealed significant age‐related functional changes across various cell types, including stem cell exhaustion, FAP functional imbalance, and an increased proportion of immune cells (Antuña et al. 2022; Lopez‐Otin et al. 2023; Theret et al. 2021). We selected LDM and PMM, which do not exhibit a clear metabolic bias, to compare age‐related differences in the same type of muscle nuclei (e.g., type II muscle nuclei) across different tissues. With aging, the overall composition of the fundamental cell types in LDM and PMM remains relatively stable. Notably, major cell subtypes exhibited significant expansion with age. Consistent with prior research, the number of type II muscle nuclei decreased with age, whereas type I muscle nuclei increased relatively (Murgia et al. 2017). Notably, the RNA splicing pathway is significantly enriched in myonuclear subtypes. Consistent with this, we identified some known gene isoforms, such as *Ryr1* (associated with skeletal muscle atrophy (Tang et al. 2015)), *Tnnt3* (related to aging (Coble et al. 2015)), and *Mef2c* (linked to myogenic activity (Zhang et al. 2015)), and also observed alternative splicing in other gene isoforms, including *Atp2a1*, *Gapdh*, and *Slc25a4*, which are associated with mitochondrial function. This highlights the critical role of alternative splicing in the muscle aging process, particularly in the regulation of gene expression related to energy metabolism and muscle function.

Our study also identified rare cellular states, such as the depletion of IIb‐2 and IIb‐3 muscle nuclei subtypes. IIb‐2 muscle nuclei marker genes were significantly enriched in the PI3K/AKT signaling pathway, a key regulatory axis in phosphatidylinositol (PI) signaling that promotes protein synthesis and muscle cell growth. This pathway is necessary for muscle hypertrophy and preventing muscle atrophy, aligning with findings that aging‐induced atrophy primarily affects fast‐twitch muscle fibers (Akasaki et al. 2014; Liu et al. 2023; Mishra et al. 2022; Purves‐Smith et al. 2014; Shi et al. 2023). Furthermore, we identified atrophy‐related genes such as *Cdkn1a*, *Ankrd1*, and *Gadd45a*, which were uniquely significantly upregulated in aging type IIb‐2 myonuclei (Reedich et al. 2021; Wu et al. 2024; Xu et al. 2024). This suggests that type IIb‐2 myonuclei may play a key role in age‐related muscle atrophy.

IIb‐3 myonuclei, in contrast, exhibited high metabolic activity. Previous studies classified this subtype as the IIx‐b cell population, characterized by high expression of *Myh1* and *Myh4*, rather than a transitional state between cluster IIx and IIb (Chemello et al. 2020). Consistent with this classification, we identified IIb‐3 at the terminal stage of the IIB myonuclear dynamic trajectory, with a significant downregulation of oxidative phosphorylation‐related genes during aging. High oxidative phosphorylation levels are typically associated with better mitochondrial function in myofibers, whereas mitochondrial function in glycolytic (type II) muscle fibers declines significantly with aging (Crupi et al. 2018; Mishra et al. 2015; Sin et al. 2016). Our results indicate that the oxidative phosphorylation downregulation in type II myofibers is primarily concentrated in IIb‐3, as evidenced by the decreased percentage of IIb‐3 myonuclei and the downregulation of oxidative phosphorylation‐related genes. These findings highlight the myonuclear subclusters as potential targets for therapeutic interventions aimed at mitigating muscle aging. Notably, the IIb‐type myonuclear subpopulations (IIb‐1, IIb‐2, IIb‐3) identified in the mouse model do not directly correspond to human muscle fiber types. Therefore, the translational relevance of these findings to human aging processes is limited.

The differential responses of skeletal muscle tissues to aging are not entirely dependent on fiber‐type composition, suggesting that other cell types may contribute to aging‐related changes (Lai et al. 2024; Ma et al. 2020). With aging, non‐muscle cell populations within the skeletal muscle microenvironment undergo significant transcriptional shifts, exhibiting high heterogeneity. Notably, adipocytes transition from a lipid metabolism‐related subtype to one characterized by pathways involved in cell adhesion and neurodegenerative diseases, accompanied by the emergence of a small cluster of *Ucp1*‐positive adipocytes. This aligns with studies showing that aging induces myosteatosis, a pathological fat accumulation in skeletal muscle, leading to lipid infiltration (Wang, Valencak, and Shan 2024). Interestingly, *Ucp1*‐positive adipocytes are typically found in brown adipose tissue, where they promote thermogenesis and counteract obesity (Lundgren et al. 2023). However, our findings reveal an increase in *Ucp1*‐positive adipocyte clusters within aging skeletal muscle. Other studies confirm that intermuscular adipose tissue (IMAT) in both humans and mice contains adipocytes with characteristics similar to *Ucp1*‐positive brown fat cells (Almind et al. 2007; Crisan et al. 2008; Zhang et al. 2023). Furthermore, mice engineered to ectopically express *Ucp1* in skeletal muscle exhibit extended lifespans and resistance to metabolic dysfunction, suggesting a potential role in promoting healthy aging (Keipert et al. 2010; Masania et al. 2023). Our results highlight that ectopic *Ucp1* expression in skeletal muscle is closely related to adipocyte plasticity and functional changes, which may contribute to maintaining metabolic health during aging.

FAPs play crucial roles in muscle fibrosis and steatosis, with fibrosis specifically characterized by excessive accumulation of extracellular matrix (ECM) proteins (Wang et al. 2023). Our results indicate that myofibroblasts are located at the end of the trajectory, and studies have shown that myofibroblasts exhibit contractility similar to that of smooth muscle cells, participating in the fibrotic process (Plikus et al. 2021). Consistent with this, several fibrosis‐related genes, such as *Fbn1* and procollagen genes (*Col1a1*, *Col1a2*, and *Col3a1*), exhibited significant changes (Li et al. 2024; Miyauchi et al. 2024). In contrast, we did not observe any significant changes in the expression of adipogenic‐related genes. This suggests that during muscle aging, FAPs are more inclined towards fibrosis than adipogenesis. Additionally, the ECM pathway is significantly enriched in both *Lum* + FAP and *Fbn1* + FAP, indicating an increase in ECM protein deposition, which may further lead to skeletal muscle stiffness or fibrosis during the injury repair process, impairing the repair and regeneration of skeletal muscle (Lukjanenko et al. 2019).

Aging was associated with an increase in B cells and T cells, consistent with previous findings in the aging mouse brain, liver, and adipose tissue, as well as with age‐related immune infiltration (Dulken et al. 2019; Mangiola et al. 2023; Schaum et al. 2020). In addition, we identified the characteristics of four macrophage subpopulations, with immune activation pathways significantly enriched in PVM and NPVM, the two predominant subpopulations. PVM expressed high levels of *Lyve1* and exhibited M2‐type characteristics, contributing to tissue repair (Cui et al. 2023; Sárvári et al. 2021). NPVM was identified as non‐vascular‐associated macrophages (Sárvári et al. 2021). These findings suggest that PVM and NPVM play important roles in immune regulation and tissue repair, particularly during aging, potentially influencing muscle degeneration and repair through modulation of inflammation and promotion of tissue remodeling.

Dysregulated intercellular communication (ICC) is a hallmark of aging, often characterized by an overrepresentation of inflammatory factors (Lagger et al. 2023; Lopez‐Otin et al. 2023; Miller et al. 2020). In this study, aged skeletal muscle exhibited increased intercellular communication, consistent with previous findings of heightened cell–cell interactions in aging skeletal muscle, particularly in inflammatory pathways (Lai et al. 2024). Notably, we observed a significant increase in interactions between Egf ligands from aged myonuclei and EGFR receptors on other cell types, leading to elevated secretion of *Il‐1b* (an inflammatory cytokine) and *Cdkn1a* (a senescence marker). Prior studies have demonstrated that EGF induces cellular senescence in IMR90 cells (normal human lung fibroblasts) via EGFR‐Ras signaling (Shang, Han, et al. 2020). Consistent with this, 50 nM EGF significantly increased the secretion of inflammatory factors and senescence markers in C2C12 cells while inhibiting myogenic factor expression. These results suggest that aging enhances intracellular communication in skeletal muscles, amplifying inflammatory factor expression in myonuclei, which may contribute to aging‐induced muscle atrophy. It is important to note that the H_2_O_2_‐induced oxidative stress model used in this study, while widely employed for investigating age‐related cellular damage, does not fully replicate the natural aging process or all its physiological aspects. These limitations should be considered when interpreting the findings.

Currently, most research on muscle aging mechanisms focuses on male animal models (De Masi et al. 2024; Petrany et al. 2020; Zhang et al. 2023). However, skeletal muscle structure and function exhibit a high degree of sex specificity, with nearly 3000 genes showing differential expression between sexes (Oliva et al. 2020). Therefore, it is inappropriate to generalize findings from male studies to female populations without considering the sex‐specific aspects of skeletal muscle aging. In fact, females generally have a longer lifespan than males (Murtagh and Hubert 2004). Although the processes of muscle aging in both males and females are similar, the extent of changes in these pathways differs between the sexes (de Jong et al. 2023). Therefore, we believe that exploring the aging mechanisms of different muscle tissues in female mice provides a clearer foundation for future research, particularly in the area of sex‐specific muscle aging mechanisms.

We acknowledge that the lack of a comprehensive sex comparison is a limitation of this study. By focusing exclusively on female mice, we aimed to minimize confounding variables, such as sex‐specific hormone levels, which could complicate result interpretation. However, this may limit the generalizability of our findings across both sexes. Future studies will include male mice for a comparative analysis to better understand the impact of sex on muscle aging mechanisms.

## 4 Conclusions

In conclusion, our study underscores the complex and tissue‐specific nature of skeletal muscle aging. By revealing age‐related changes across distinct skeletal muscle types and cellular subpopulations, we provide new insights into the molecular mechanisms driving muscle aging and its associated pathologies. These findings establish a foundation for future therapeutic strategies aimed at combating muscle aging and improving the quality of life in aging populations.

## 5 Methods

### 5.1 Animals and Sample Collection

All animal experiments were approved by the Institutional Animal Care and Use Committee of the College of Animal Science and Technology, Sichuan Agricultural University, China (Approval number: 20230625). Female C57BL/6J mice were housed under a 12‐h light/dark cycle with ad libitum access to standard laboratory chow. All experimental mice were of specific pathogen‐free (SPF) grade. Mice were sacrificed at 3 and 24 months of age, and 11 tissues were collected, including ventricle, diaphragm, extensor digitorum longus (EDL), masseter, soleus, gastrocnemius, quadriceps, tibialis anterior (TA), longissimus dorsi muscle (LDM), psoas major muscle (PMM), and semitendinosus (ST). The tissues were immediately flash‐frozen in liquid nitrogen. To minimize variation, the sample collection process was carefully optimized.

### 5.2 RNA Sequencing and Analysis

PolyA‐enriched RNA‐seq libraries were constructed and sequenced on the Illumina NovaSeq 6000 platform, generating an average of ~43 million 150‐bp paired‐end raw reads per library, with ~41 million high‐quality reads retained. Sequencing reads were aligned to the mouse reference genome (GRCm39) using STAR (version 2.5.3a) and mRNA expression levels were quantified with Kallisto (version 0.44.0). Genes with TPM (Transcripts per million) > 0.5 in at least one sample are considered detectable, yielding 16,945 qualified genes across the examined tissues. DEG analysis was conducted using the edgeR package in R, with DEGs defined as those with |Log_2_FC| > 1 and a significance false discovery rate (FDR) < 0.05.

### 5.3 Total RNA Extraction and Quantitative Reverse Transcription PCR (RT‐qPCR)

Total RNA was extracted using TRIzol reagent (Invitrogen) following the manufacturer's protocol. Reverse transcription was performed using a HiScript III RT SuperMix for qPCR (+gDNA wiper) kit (Vazyme) according to the manufacturer's instructions. RT‐qPCR was conducted on at least three independent biological samples using ChamQ Universal SYBR qPCR Master Mix (Vazyme) on a CFX96 Real‐Time PCR System (Bio‐Rad). Relative gene expression levels were calculated using the 2^−ΔΔCt^ method, with β‐actin as the internal control. The primer sequences used in this study are listed in Table S6.

### 5.4 Immunofluorescence, Succinate Dehydrogenase (SDH) Staining, and Hematoxylin and Eosin (HE) Staining

For immunofluorescence, tissue sections were air‐dried at 37°C for 10–20 min, fixed in fixative for 30 min, and washed with PBS (pH 7.4). Antigen retrieval was performed under standard conditions, followed by cooling and washing. Endogenous peroxidase activity was blocked with 3% hydrogen peroxide for 25 min, and non‐specific binding was blocked with 10% rabbit serum or 3% BSA for 30 min. Sections were incubated overnight at 4°C with primary antibodies, followed by a 50‐min incubation with HRP‐conjugated secondary antibodies. TSA amplification was performed, followed by microwaving for antigen retrieval. A second round of antibody incubation was conducted using fluorescent secondary antibodies, and nuclei were counterstained with DAPI. Fluorescence images were captured using appropriate excitation and emission wavelengths.

For SDH staining, muscle tissues embedded in OCT were sectioned into 10 μm thick frozen sections. Sections were stained using a SDH Staining Kit (Solarbio, Cat# G2000) at 37°C for 20 min, followed by a deionized water wash. Stained sections were imaged under a microscope, and muscle fiber diameters were measured using ImageJ software.

For HE staining, tissue sections were dewaxed and rehydrated using Dewaxing Solution I and II for 20 min each, followed by sequential dehydration in anhydrous ethanol I and II (5 min each) and 75% ethanol (5 min), with a final water rinse. After rinsing, sections were differentiated, blued, and rinsed again. Eosin staining was performed for 15 s following a 95% ethanol wash. Samples were dehydrated through a graded series of anhydrous ethanol, tert‐butanol, and xylene (2 min each) and mounted with neutral resin. Microscopic observation and imaging were then conducted.

The antibodies used for immunofluorescence are listed in Table S7.

### 5.5 Hanging Test

The hanging time was measured according to the method described by Shang, Sun, et al. (2020). A 45 × 45 cm grid (rod diameter: 2 mm; grid spacing: 18 mm) was mounted on a frame 55 cm above the ground, with a 5 cm thick cushion placed underneath. The distance between the grid and the cushion was maintained at 50 cm. Each mouse was placed in the center of the grid, which was then inverted, positioning the mouse head‐down. The duration for which the mouse was able to hold on before falling was recorded. Each mouse underwent three trials with a 30‐min interval between tests. The average hanging time was calculated.

### 5.6 Skeletal Muscle Nuclei Isolation

Fresh skeletal muscle was stored in GEXSCOPE Tissue Preservation Solution (Singleron Biotechnologies, Nanjing, China) at 4°C until library preparation. Nuclei were isolated using the GEXSCOPE Nuclear Isolation Solution (Singleron Biotechnologies, Nanjing, China) following the manufacturer's instructions. Nuclei from the same tissue (*n* = 6) were pooled and resuspended to a concentration of 10^6^ nuclei per 400 μL in PBSE, filtered through a 40 μm cell strainer, and counted using trypan blue. Nuclei were enriched in PBSE and stained with DAPI (1:1000 dilution, Thermo Fisher Scientific, D1306), with DAPI‐positive singlets defined as nuclei.

### 5.7 Single‐Nucleus RNA‐Sequencing Library Preparation

The concentration of the single nucleus suspension was adjusted to 3–4 × 10^5^ nuclei/mL in PBS. This suspension was then loaded onto a microfluidic chip (GEXSCOPE Single Nucleus RNA‐seq Kit, Singleron Biotechnologies), and snRNA‐seq libraries were constructed following the manufacturer's instructions. The libraries were sequenced using paired‐end 150 bp sequencing mode on the DNBSEQ‐T7 platform.

### 5.8 Data Processing, Dimensional Reduction, Cell Type Identification, and Differential Expression Genes Analysis

Raw reads from snRNA‐seq were processed to generate gene expression matrices using the CeleScope pipeline (https://github.com/singleron‐RD/CeleScope). The environmental RNA background for each library was assessed and corrected using the R package SoupX (Young and Behjati 2020). Subsequent visualizations, clustering, and differential expression analyses were performed in R (v4.1.3) using Seurat (v4.4.0). Quality control was aligned to retain cells with > 200 and < 5000 nFeature RNA, > 500 and < 10,000 total counts, and < 5% mitochondrial gene content. After filtering, a total of 73,170 nuclei were obtained, with an average of approximately 700–800 genes expressed per nucleus. The expression matrix was then integrated, and dimensionality was reduced using the RunUMAP function for clustering in two‐dimensional space. The FindAllMarkers function in Seurat was used for unbiased marker gene identification. Cluster identities were annotated based on known marker gene expression patterns described in the literature. DEGs were identified using the FindMarkers function, with |Log_2_FC| > 0.25 and an adjusted *p*‐value < 0.01 considered statistically significant. Marker genes for each cell type are listed in Table S4.

### 5.9 Pseudotime Analysis

Pseudotime analysis was performed using Monocle3 to infer developmental trajectories. After dimensionality reduction with UMAP, Monocle3's trajectory inference algorithm was applied with default parameters to order cells along pseudotime. CytoTRACE was used to define the dynamic trajectory starting point. CytoTRACE scores were calculated based on gene expression profiles, with values ranging from 0 to 1‐higher scores indicating a more stem‐like (less differentiated) state and lower scores indicating greater differentiation. Based on the annotated cell types, the differentiation results helped identify the root cell for the pseudotime trajectory.

### 5.10 Intercellular Interactions

Cell–cell interaction analysis was performed using the CellChat package (v1.6.1), which infers communication networks from single‐cell RNA sequencing data. The input data consisted of the normalized gene expression matrix from the snRNA‐seq. CellChat identified signaling pathways by analyzing ligand‐receptor interactions and visualized significant communication networks within the cellular microenvironment. Cell types were annotated based on known markers, and all analyses were conducted using CellChat's default parameters.

### 5.11 Transcription Factor Analysis

Transcription factor activity was assessed using the SCENIC (Single‐Cell Regulatory Network Inference and Clustering) package (v0.12.1). The SCENIC workflow was applied to infer gene regulatory networks and evaluate transcription factor activity at the single‐cell level. Input data consisted of the normalized gene expression matrix from snRNA‐seq. SCENIC identified co‐expressed gene modules and their corresponding transcription factors, generating potential regulons—transcription factors regulating specific gene sets. The SCENIC output was analyzed to determine transcription factor activity variations across different cell states.

### 5.12 Alternative Splicing Analysis

We performed alternative splicing analysis at single‐cell resolution using the MARVEL package (MARVEL: an integrative alternative splicing analysis platform for single‐cell RNA sequencing data). This tool supports both droplet‐based and plate‐based single‐cell splicing analysis. We utilized gene expression and splice junction count matrices generated by STARsolo as inputs for MARVEL. The software quantified the Percent Spliced‐In (PSI) values for all seven major types of exon‐level alternative splicing events and identified differentially spliced events. All analyses were conducted using MARVEL's default parameters.

### 5.13 Cell Culture and Drug Treatment

The C2C12 myoblast cell line was obtained from the Cell Bank of the Chinese Academy of Sciences, Shanghai (https://www.cellbank.org.cn/). Control cells were cultured in DMEM supplemented with 10% FBS and 1% penicillin–streptomycin at 37°C in a 5% CO_2_ incubator. When cells reached 60%–70% confluence, the medium was replaced with DMEM containing 2% horse serum to induce differentiation. The differentiation medium was refreshed every other day, and cells were collected for further experiments on Day 6 when distinct myotube formation was observed.

For the treatment group, cells (5 × 10^4^) were seeded in 12‐well flat‐bottomed plates. On the second day of differentiation induction, the medium was replaced with a differentiation medium containing various concentrations of reagents. Cells were cultured for 4 days, with medium changes every 2 days. The reagents included H_2_O_2_ (485 μM/mL), gefitinib (100 ng/mL), and different concentrations of EGF. All experiments were performed using cells passaged no more than eight times to prevent false positives caused by high‐passage effects (Rahman et al. 2024).

## Author Contributions

C.L., D.Z., and R.Z. contributed equally to this work. L.J. designed the experiments. X.T., Y.S., C.W., and D.L. performed animal work and prepared biological samples. Y.W., G.Z., H.C., and Z.R. conducted phenotypic experiments. D.Z. and H.L. designed the bioinformatics analyses. C.L. and D.Z. performed the data analysis. R.Z. conducted cell culture experiments. S.W. conducted the gene quantification experiment. C.L. drafted the paper. L.J., X.L., and M.L. revised the paper. L.J. acquired the funding. All authors have read and agreed to the published version of the manuscript.

## Conflicts of Interest

The authors declare no conflicts of interest.

## Supporting information


Appendix S1.

Appendix S2.

## Data Availability

The RNA‐seq data supporting the findings of this study have been deposited in NCBI's GEO under accession number GSE287451, and the snRNA‐seq data is available in the Sequence Read Archive under accession number PRJNA1211289.
