# Supplementary material for: Transcriptional Diversity in Response to Aging Across Skeletal Muscles
Source: Aging Cell. 2025 Jul 9;24(9):e70164. doi: 10.1111/acel.70164 (PMC12419864; doi:10.1111/acel.70164)
Supplement: Supplementary file 1 — Appendix S1. [file ACEL-24-e70164-s002.docx]

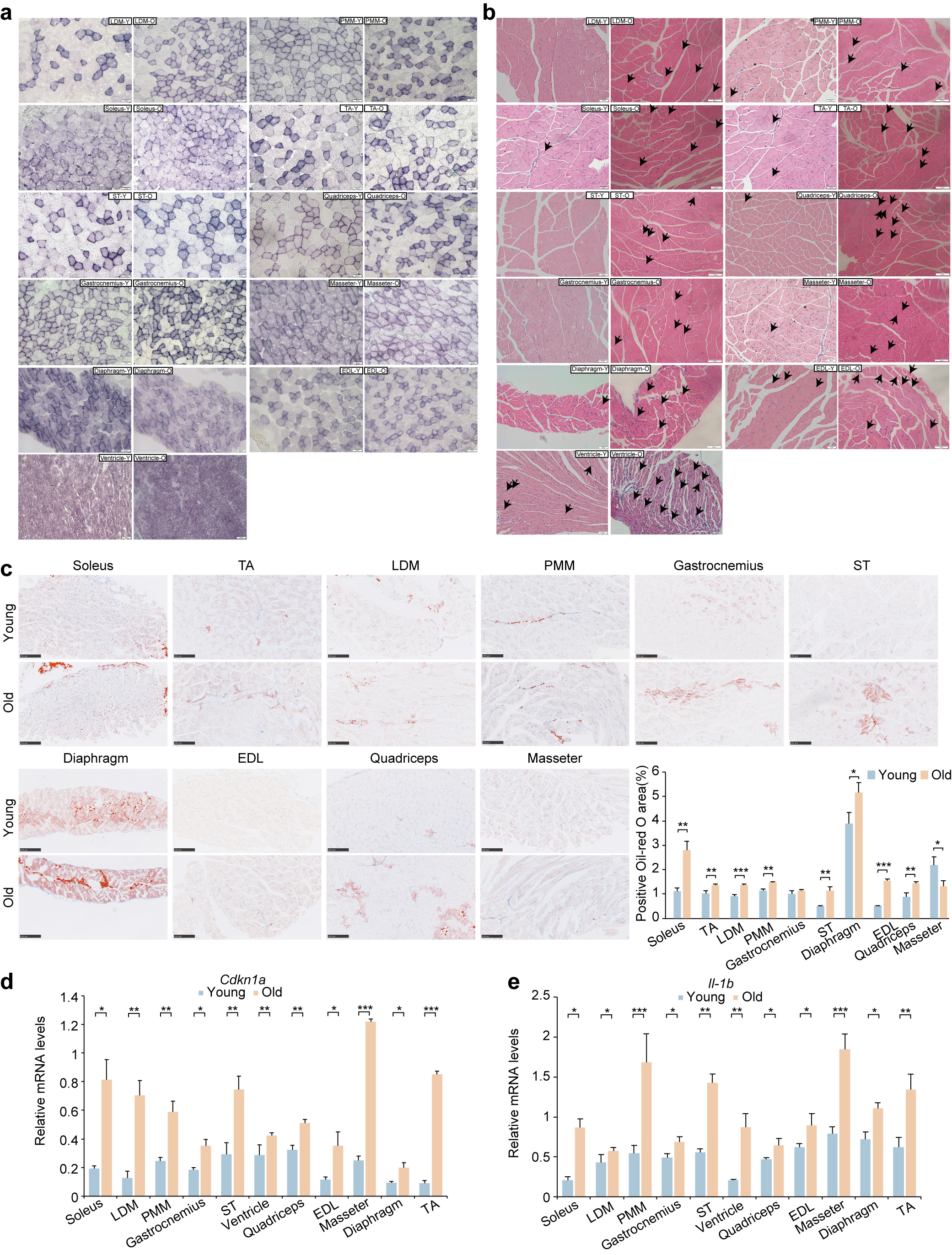


**Figure S1. Phenotypic and PCR analysis of skeletal muscle in young and old mice**

**(a)** Representative images of SDH staining demonstrating muscle fiber diameter in various tissues. Scale bar = 50 μm.

**(b)** Hematoxylin-eosin staining (HE) shows a muscle fiber with a central nucleus (arrows). Scale bar = 50 μm.

**(c)** Oil Red O staining shows muscle fat infiltration. Positive Oil Red O area presented as mean ± SD. **p < 0.05,* ***p < 0.01,* ****p < 0.001.*

**(d, e)** Relative expression of inflammatory (*P21*) **(c)** and aging (*IL-1β*) **(d)** marker genes validated by qRT-PCR in 11 tissues. Data are presented as mean ± SD. **p < 0.05,* ***p < 0.01,* ****p < 0.001.*


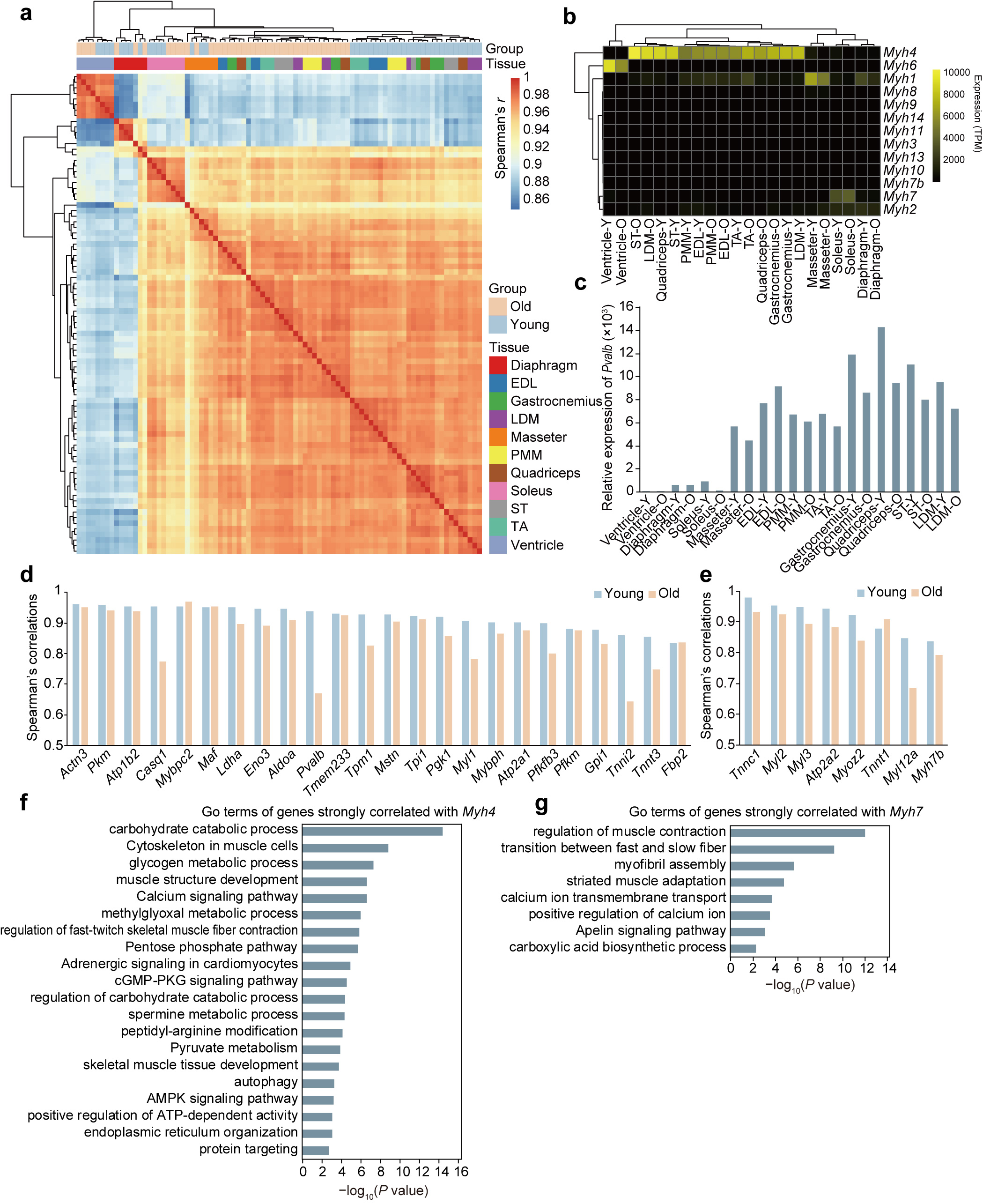


**Figure S2. Gene expression correlation and functional analysis in skeletal muscles of young and old mice**

**(a)** Correlation (Spearman) heatmap of mRNA expression across 11 different tissues in young and old mice.

**(b)** Clustering heatmap of the absolute expression of Myosin heavy chain (Myh) transcripts across 11 skeletal muscle tissues in young and old mice.

**(c)** The TPM expression of *Pvalb* is shown as a bar graph.

**(d, e)** Bar graph showing correlation coefficients of *Myh4* **(d)** and *Myh7* **(e)** with other genes. Only genes with an *R* > 0.8 are shown.

**(f, g)** GO and KEGG enrichment analysis of genes correlated with *Myh4* (*R* > 0.8) and *Myh7* (*R* > 0.8), separately.


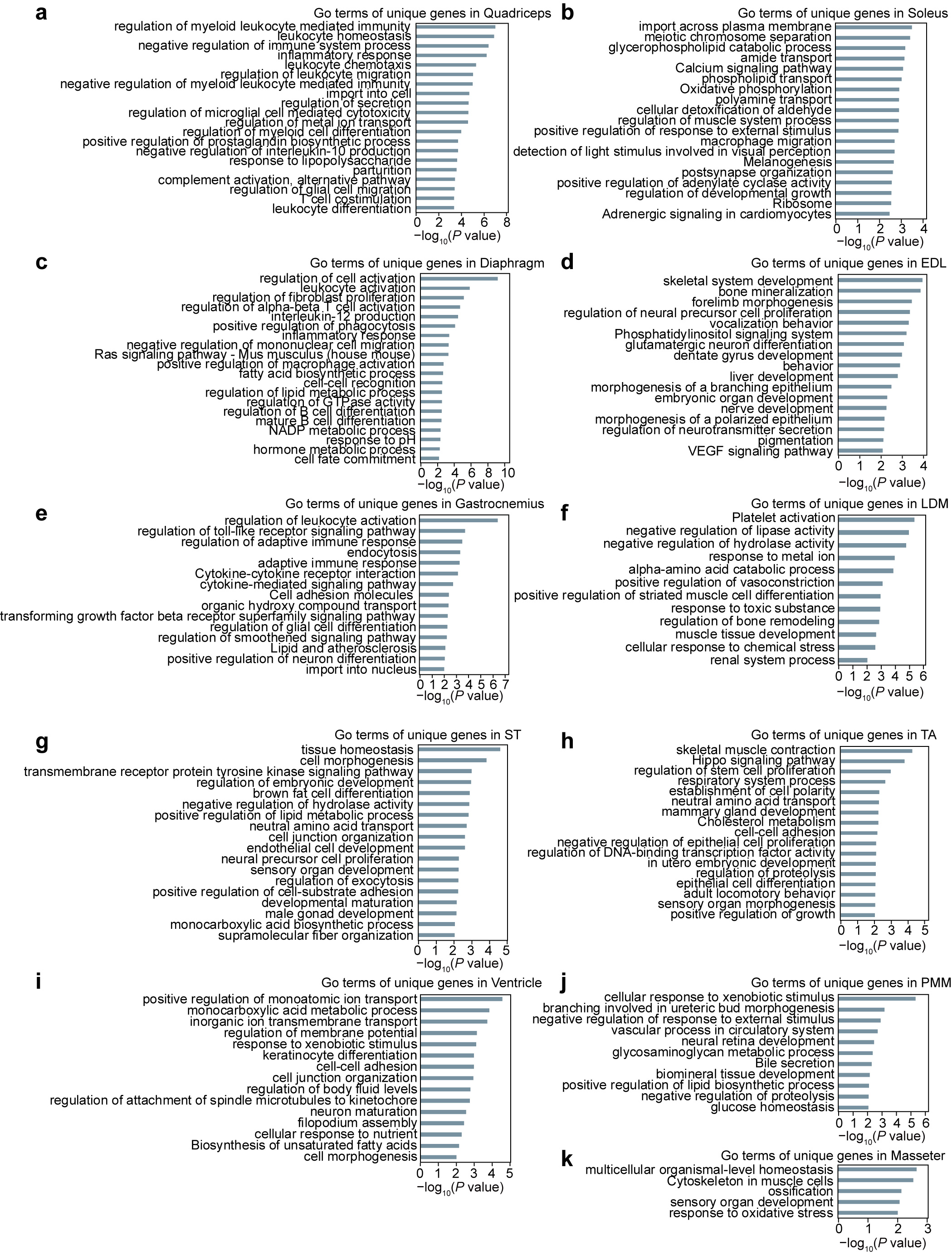


**Figure S3.** **GO and KEGG enrichment analysis of unique genes across 11 tissues**

(a-k) Enrichment analysis of genes specific to each tissue, showing significant GO and KEGG pathways.


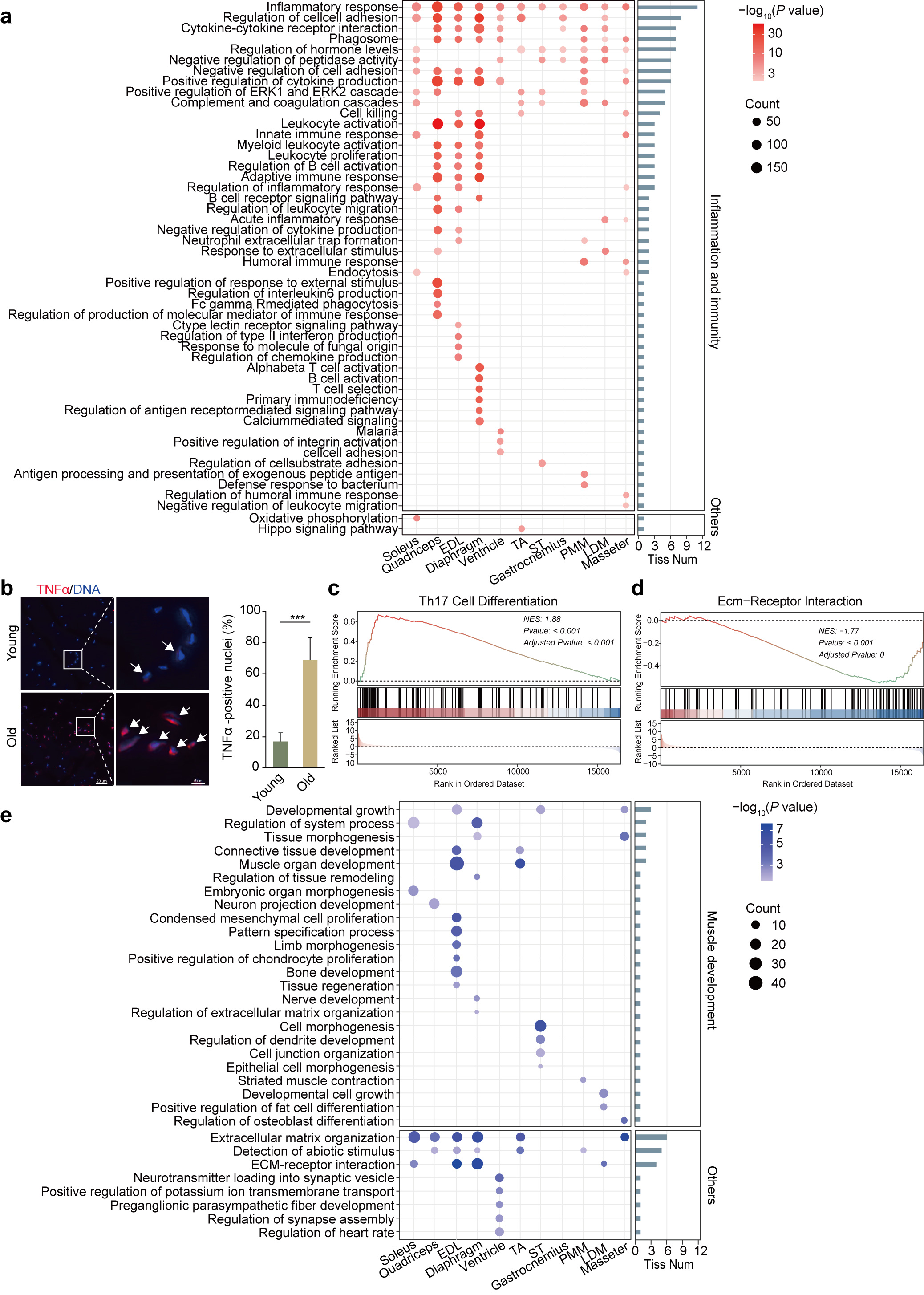


**Figure S4** **Functional analysis of gene expression and pathways in different tissues**

**(a)** Representative GO terms and KEGG pathways of up-regulated genes across all tissues. Histogram indicates the number of tissues with the same enrichment pathway.

**(b)** Representative immunostaining images of TNFa in the young and old groups are shown on the left. Scale bar, 20 and 10 μm (zoomed-in image). Gene immunofluorescence intensities were quantified and presented as mean ± SD on the right. ****p* < 0.001.

**(c, d)** Gene set enrichment analysis (GSEA) of the **(c)** Th17 cells differentiation and ECM-receptor interaction **(d)** in Dia. NES: normalized enrichment score.

**(e)** Representative GO terms and KEGG pathways of down-regulated genes across all tissues. Histogram indicates the number of tissues with the same enrichment pathway.


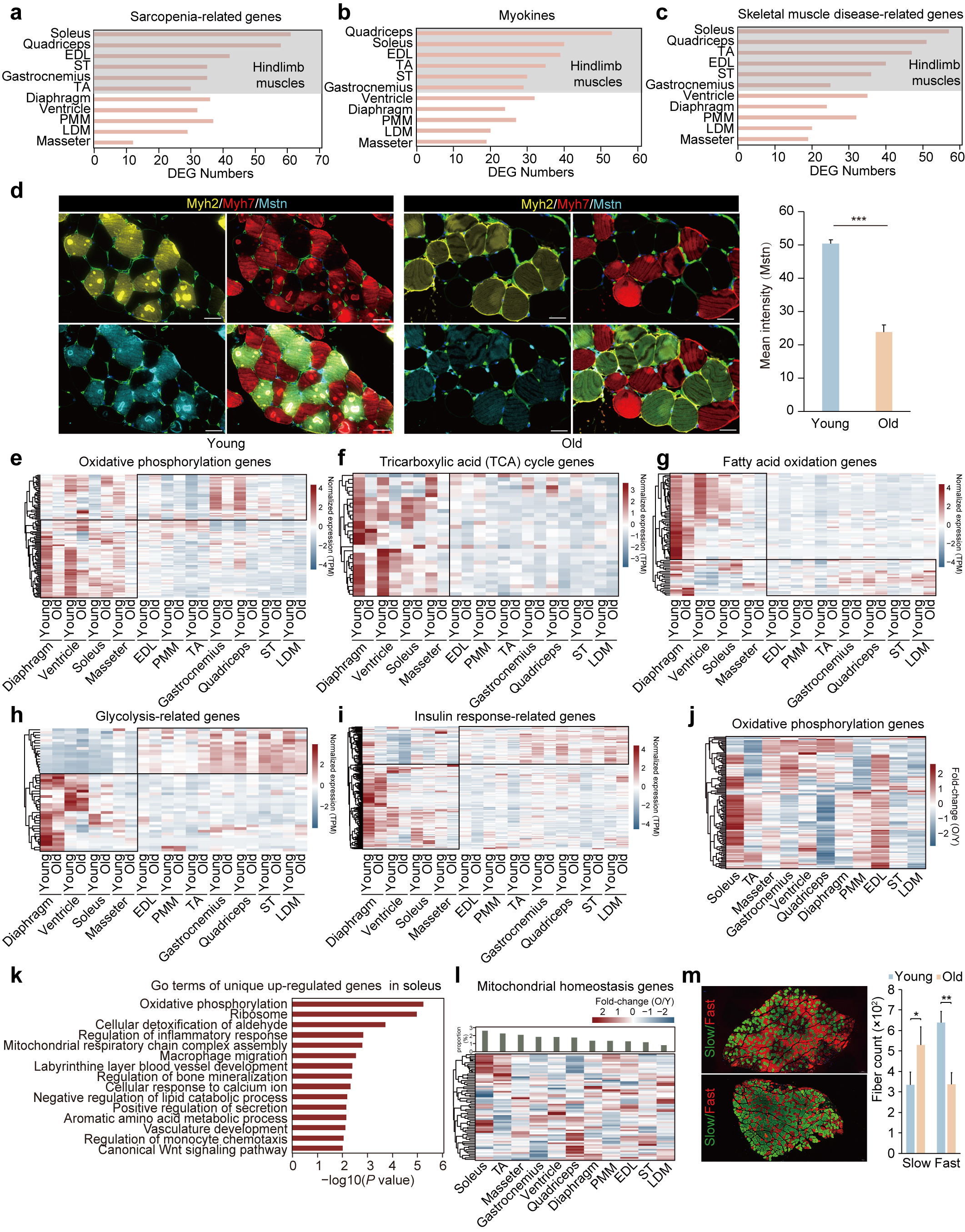


**Figure S5. Tissue-specific alterations of DEGs related to muscle diseases and metabolism**

**(a-c)** Bar graph depicting the number of differentially expressed genes (DEGs) related to sarcopenia **(a),** myokines **(b)** and skeletal muscle disease **(c)** across various tissues.

**(d)** Representative immunostaining images of Myh2, Myh7 and Mstn in the young and old groups. Scale bar = 20 μm. Mstn immunofluorescence intensities were quantified and presented as mean ± SD on the right. ****p* < 0.001.

**(e-i)** Normalized expression of gene transcripts involved in oxidative phosphorylation **(e)**, TCA cycle **(f)**, fatty acid oxidation **(g)**, glycolysis **(h)**, and response to insulin **(i)**, represented as a heatmap. The overall similarity in gene expression across tissues is represented as a dendrogram (left).

**(j)** A heatmap illustrating fold changes in gene expression related to oxidative phosphorylation.

**(k)** Representative GO terms and KEGG pathways of unique up-regulated genes in soleus.

**(l)** A heatmap showing fold changes in gene expression linked to mitochondrial homeostasis. The bar chart (top) represents the percentage distribution of mitochondrial homeostasis-related genes among DEGs in various tissues.

**(m)** Cross-sections of skeletal muscle stained with immunofluorescence-specific antibodies for fast II and slow I muscle fibers. Representative images are shown on the left, with the scale bar indicating 100 μm. The number of fast II and slow I muscle fibers is shown on the right as mean ± SD. **p* < 0.05, ***p* < 0.01.


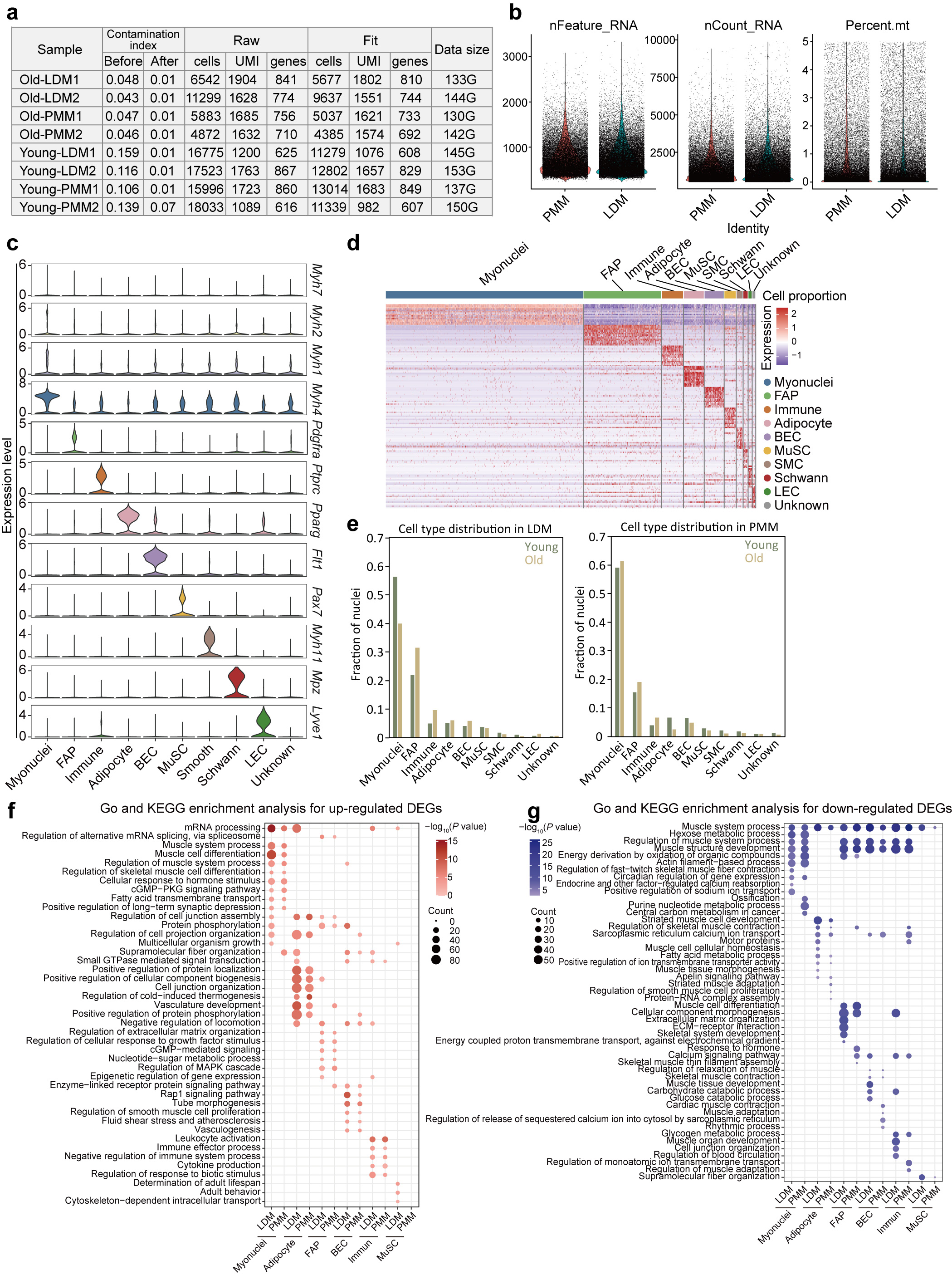


**Figure S6 Comprehensive analysis of sequencing quality, gene expression, and cell-type-specific pathways**

**(a)** Mapping statistics of sequencing results of each sample before and after quality control.

**(b)** Violin plots showing the number of detected genes (nFeature), total reads (nCount), and the Percentage of mitochondrial genes (percent_mt) in each tissue.

**(c)** Violin plots showing the expression of marker genes in the different cluster.

**(d)** Normalized expression heatmap showing the top 15 marker genes for each cluster of nuclei.

**(e)** Distribution of cell types in LDM (left) and PMM (right) for young and old groups, with the fraction (relative to the total number of nuclei) of each cell type shown on the y-axis.

**(f, g)** Dot plot summarizing common GO terms and KEGG pathways enriched among up-regulated (F) and downregulated (G) DEGs in major cell types of LDM and PMM.


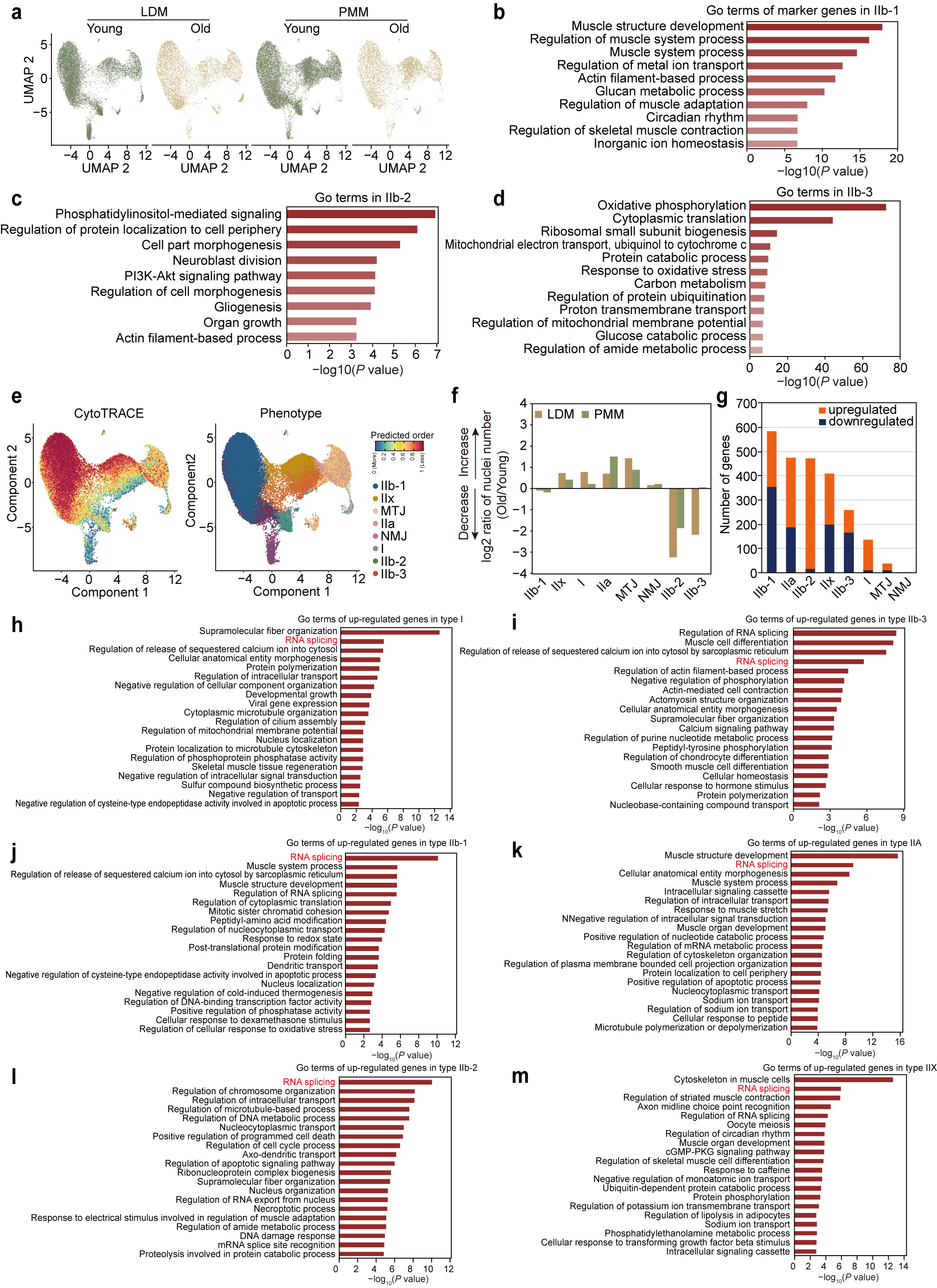


**Figure S7 Cellular heterogeneity and functional analysis across young and old mouse skeletal muscles**

**(a)** UMAP diagram visualized nuclear cluster of different tissues in the young and old groups.

**(b-d)** Representative GO terms and KEGG pathways of marker genes in type IIb subclusters.

**(e)** CytoTRACE pseudotime differentiation trajectory (left), UMAP visualization showing the mapped phenotype information (right).

**(f)** The bar chart shows the log2 ratio of the fraction (Old/Young) for each muscle fiber type, calculated as the proportion of each cell type relative to the total number of nuclei in LDM and PMM. Positive values indicate an increased fraction of the cell type in the old group compared to the young group, while negative values represent a decreased fraction.

**(g)** The number of upregulated and downregulated DEGs across all cell clusters. Cell types are ranked by the total number of DEGs.

**(h-m)** GO terms and KEGG pathways of up-regulated genes in major myonuclei subclusters.


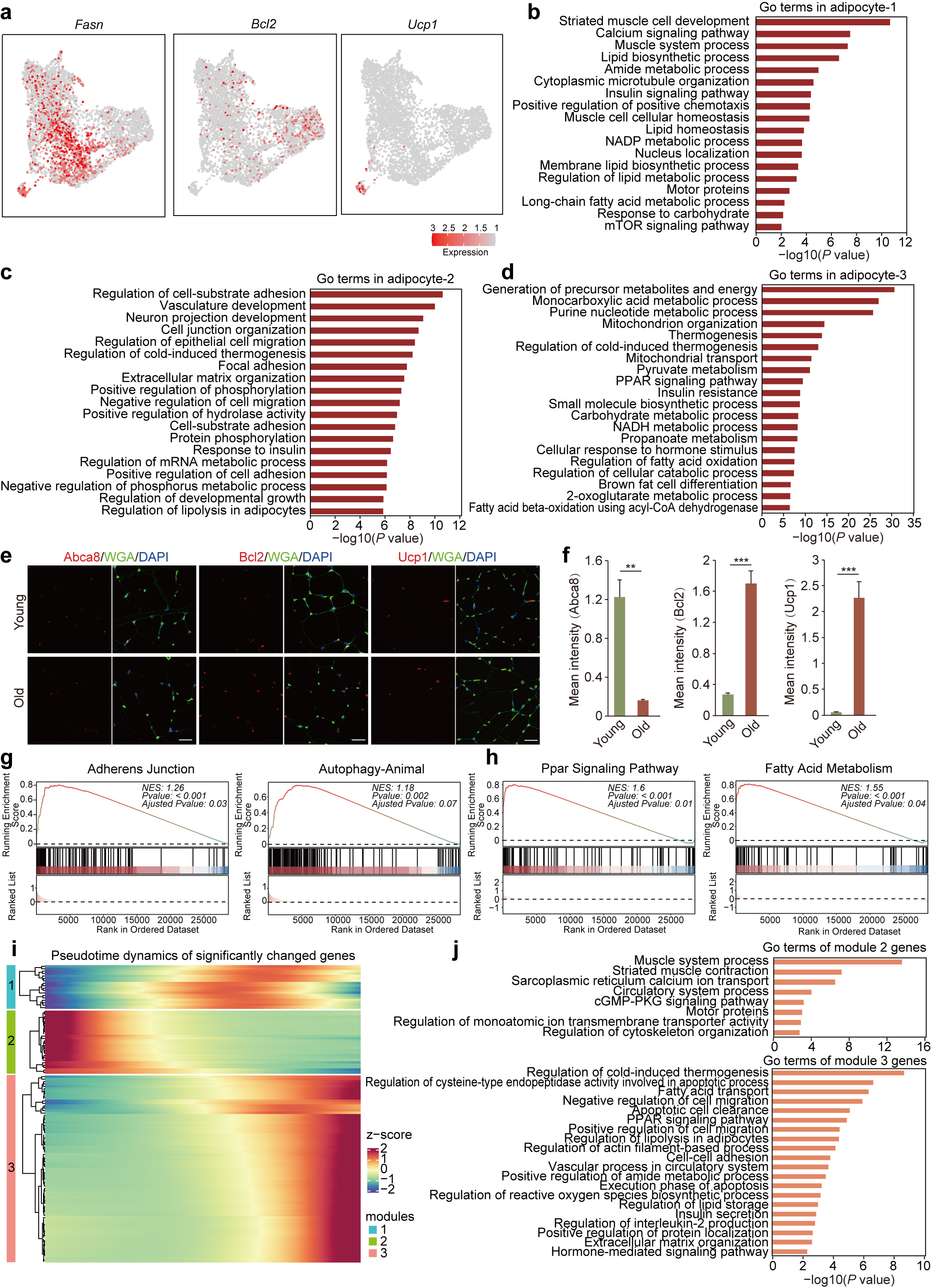


**Figure S8 Adipocyte subcluster characterization and functional pathway analysis in young and old mice**

**(a)** Umap Plots showing the expression of marker genes of adipocyte subclusters.

**(b-d)** GO terms and KEGG pathways of marker genes in major adipocyte subclusters.

**(e)** Representative immunostaining images of marker genes for adipocyte 1 (Abca8), adipocyte 1 (Bcl2) and adipocyte 3 (Ucp1) in the young and old groups. Scale bar = 20 μm.

**(f)** Marker gene immunofluorescence intensities were quantified and presented as mean ± SD. ***p* < 0.01, ****p* < 0.001.

**(g, h)** Gene set enrichment analysis (GSEA) in adipocyte2 (G) and adipocyte3 (H), NES: normalized enrichment score.

**(i)** Pseudotemporal heat map showing gene expression dynamics for significant marker genes.

**(j)** GO terms and KEGG pathways of genes in Modules 2 and 3.


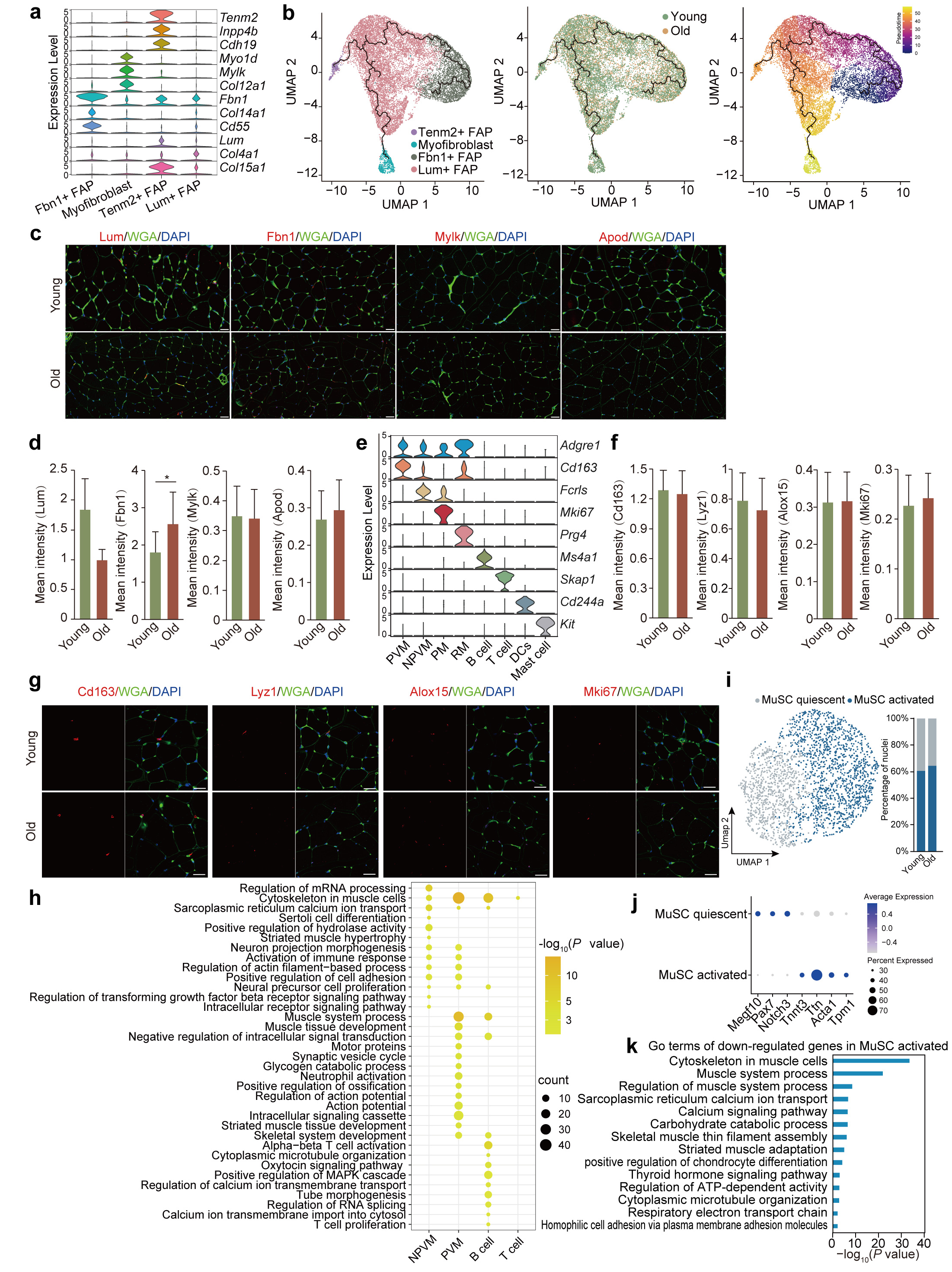


**Figure S9 Characterization and functional analysis of immune and FAP subclusters in young and old mice**

**(a)** Violin plots showing the expression of marker genes in the different cluster.

**(b)** UMAP showing the trajectory of FAP subclusters obtained from young and old groups (left). The nuclei are colored according to groups (middle) and pseudotime (right).

**(c)** Representative immunostaining images of marker genes for Lum^+^ FAP (Lum), Fbn1^+^ FAP (Fbn1), Myofibroblast (Mylk) and Tenm2^+^ FAP (Apod) in the young and old groups. Scale bar = 20 μm.

**(d)** Marker gene immunofluorescence intensities were quantified and presented as mean ± SD on the right. **p* < 0.05.

**(e)** Violin plots showing the expression of marker genes in the different cluster.

**(f)** Marker gene immunofluorescence intensities were quantified and presented as mean ± SD.

**(g)** Representative immunostaining images of marker genes for PVM (Cd163), NPVM (Lyz1), RM (Alox15) and PM (Mki67) in the young and old groups. Scale bar = 20 μm.

**(h)** Dot plot of GO terms and KEGG pathways enriched in major Immune cell subcluster.

**(i)** Umap diagram of MuSC subclusters (left). The proportion of each MuSC subclusters in young and old groups (right).

**(j)** Dotplot of marker genes in MuSC subclusters.

**(k)** GO terms and KEGG pathways of down-regulated genes in the MuSC activated subcluster.


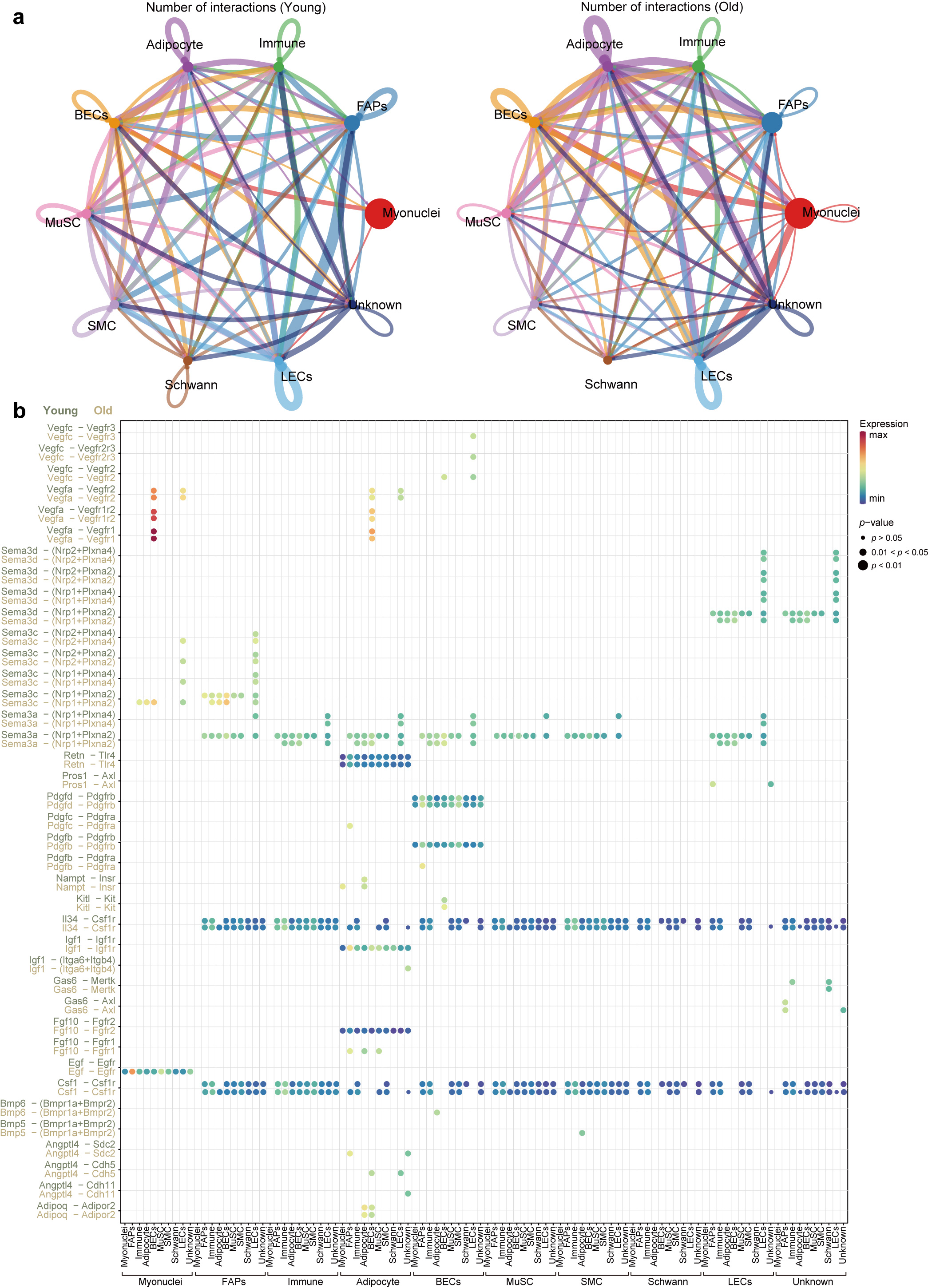


**Figure S10 Cell-cell interaction network and ligand-receptor specificity changes between young and old groups**

**(a)** Circle network plots displaying the number of interactions between cells based on the “Cellchat” package (left, young; right, old). The thickness of the lines is proportional to the number of interactions between cells. The size of the nodes is proportional to the number of different cell types.

**(b)** The ligand-receptor pairs showing significant changes in specificity between young and old groups across all cell types.


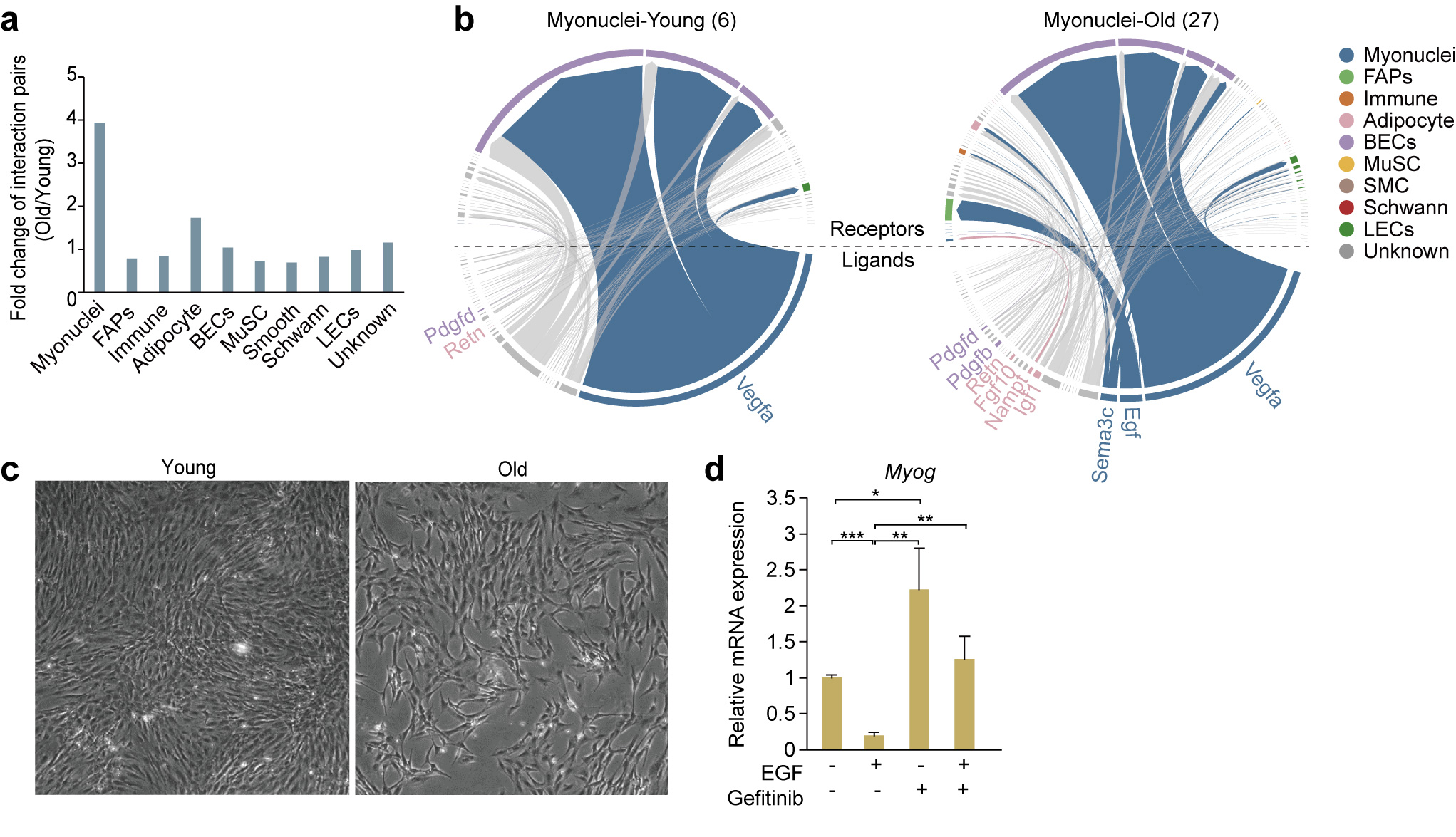


**Figure S11 Cell interaction dynamics and H₂O₂ inhibition of C2C12 differentiation**

**(a)** The vertical axis represents the fold change, indicating the ratio of the percentage of the number of interaction pairs for each cell type to the total number of interactions in the old group, divided by the same percentage in the young group.

**(c)** Chrod plot displaying intercellular ligand-receptor (L-R) interactions between myonuclei and nonmuscle nuclei in young (left) and old (right panel) groups.

**(c)** Hydrogen peroxide (H₂O₂) inhibits C2C12 differentiation, as shown by the morphological changes observed under phase contrast microscopy.

**(d)** Relative expression of *Myf5.* validated by qRT-PCR in the control group and after treatment with EGF, Gefitinib, or both.
